# CAA-derived IL-6 induced M2 macrophage polarization by activating STAT3

**DOI:** 10.1186/s12885-023-10826-1

**Published:** 2023-05-01

**Authors:** Chongru Zhao, Ning Zeng, Xiaomei Zhou, Yufang Tan, Yichen Wang, Jun Zhang, Yiping Wu, Qi Zhang

**Affiliations:** 1 Department of Plastic and Cosmetic Surgery, Tongji Hospital, Tongji Medical College, Huazhong University of Science and Technology, 1095 Jiefang Avenue, Wuhan, 430030 Hubei China; 2 Department of Thyroid and Breast Surgery, Shenzhen Qianhai Shekou Free Trade Zone Hospital, Shenzhen, 518067 Guangdong China

**Keywords:** Breast cancer, Cancer-associated adipocytes, IL-6, STAT3, Macrophages

## Abstract

**Background:**

Tumor-associated macrophages (TAMs) are the most abundant types of immune cells in the tumor microenvironment (TME) of breast cancer (BC). TAMs usually exhibit an M2 phenotype and promote tumor progression by facilitating immunosuppression. This study aimed to investigate the effect of CAA-derived IL-6 on macrophage polarization in promoting BC progression.

**Methods:**

Human BC samples and adipocytes co-cultured with 4T1 BC cells were employed to explore the properties of CAAs. The co-implantation of adipocytes and 4T1 cells in mouse tumor-bearing model and tail vein pulmonary metastasis model were constructed to investigate the impact of CAAs on BC malignant progression in vivo. The functional assays, qRT-PCR, western blotting assay and ELISA assay were employed to explore the effect of CAA-derived IL-6 on macrophage polarization and programmed cell death protein ligand 1 (PD-L1) expression.

**Results:**

CAAs were located at the invasive front of BC and possessed a de-differentiated fibroblast phenotype. CAAs facilitated the malignant behaviors of 4T1 cells in vitro, and promoted 4T1 tumor growth and pulmonary metastasis in vivo. The IHC staining of both human BC specimens and xenograft and the in vitro experiment indicated that CAAs could enhance infiltration of M2 macrophages in the TME of 4T1 BC. Furthermore, CAA-educated macrophages could enhance malignant behaviors of 4T1 cells in vitro. More importantly, CAAs could secret abundant IL-6 and thus induce M2 macrophage polarization by activating STAT3. In addition, CAAs could upregulate PD-L1 expression in macrophages.

**Conclusions:**

Our study revealed that CAAs and CAA-educated macrophages enhanced the malignant behaviors of BC. Specifically, CAA-derived IL-6 induced migration and M2 polarization of macrophages via activation STAT3 and promoted macrophage PD-L1 expression, thereby leading to BC progression.

**Supplementary Information:**

The online version contains supplementary material available at 10.1186/s12885-023-10826-1.

## Introduction

Epithelial-derived tumors primarily thrive in a dynamic stroma composed of stromal cells, immune cells, and soluble cytokines. The tumor microenvironment (TME) supplies all the necessary incentives for tumor survival, growth, and aggressiveness [1]. Macrophages are a heterogeneous group of cells in the immune system that play an important role in the body defense. Tumor-associated macrophages (TAMs) are macrophages that infiltrate into TME. In breast cancer (BC), TAMs account for more than 50% of the tumor volume and are the most abundant immune cell type in TME [2]. The number of TAMs is associated with poor prognosis in BC [3]. TAMs act in nearly the whole stage of tumor progression. Currently, two activation states of macrophages with different polarizations have been identified: classical activated (M1) and alternatively activated (M2) macrophages [4]. In the early stages of tumor development, TAMs usually exhibit an M1 phenotype with high expression of iNOS and interleukin (IL)-12, and low expression of CD206, Arg-1 and IL-10. M1 macrophages enhance the immune response to recognize and destroy cancer cells through phagocytosis and cytotoxicity, presenting anti-tumor effects. In the advanced stage of tumor progression and metastasis, TAMs usually favor the M2 phenotype, which is characterized by low expression of iNOS and IL-12, and high expression of CD206, Arg-1 and IL-10, promoting tissue repair and angiogenesis in favor of tumor progression [5, 6]. M2-type TAMs produce anti-inflammatory factors that encourage immune escape of cancer cells and further contribute to tumor progression [7].

Adipocytes are the cell type with the largest proportion in the mesenchymal stroma of BC. Studies have shown that TAMs play an essential role in the interplay between adipocytes and BC cells [8]. Adipocytes in the vicinity of BC tissue can be converted into cancer-associated adipocytes (CAAs), which promote cancer cell proliferation, enhance angiogenesis and change the extracellular matrix by secreting various cytokines such as IL-6, hepatocyte growth factor (HGF) and chemokine (C–C motif) ligand 2 (CCL2), playing an active role in the process of tumorigenesis and progression [9]. Among them, CCL2 is a common macrophage chemokines and induces M2-type macrophage differentiation, thus promoting BC progression and metastasis [10]. In addition, CAAs can secrete lactate and adenosine accumulated in the TME, and these metabolites have been shown to further induce macrophage to polarize towards M2-type [11, 12].

IL-6 mediates chronic inflammation and provides a favorable microenvironment for tumor growth. Studies have shown that circulating levels of IL-6 are correlated with the aggressive characteristics of BC patients and could lead to a worse prognosis in BC patients [13]. It was reported that IL-6 promoted the polarization of monocytes into M2-type macrophages, further enhancing the invasiveness of BC [14]. IL-6 is a strong activator of STAT3. When IL-6 binds to IL-6R and the co-receptor gp130, it activates STAT3 and the activated p-STAT3 is rapidly transferred to the nucleus, thereby activating the inflammatory cascade and oncogenic pathways [15]. STAT3 was proven to induce polarization of M2-type macrophages in ovarian cancer [16]. In gastric cancer, IL-6/STAT3 signaling could promote M2 macrophage differentiation [17]. IL-6-dependent activation of STAT3 is of importance in the progression of multiple tumors, including BC.

Recently, attention has focused on the function of CAAs in regulating immune cell recruitment. Studies on the role and mechanism of CAAs on monocytes/macrophages are not clear. Given the critical role of CAAs and TAMs in determining tumorigenesis and metastasis of cancer, we explored the interaction between CAAs, macrophages, and BC cells by utilizing a mouse BC cell line, a mouse subcutaneous tumor-bearing model and human BC tissue specimens. Our study aimed to determine the ability and the underlying mechanisms of CAAs in recruiting and polarizing macrophages to BC TME compared to normal mature adipocytes and BC cells, further affecting BC malignancy.

## Materials and methods

### Clinical BC samples

The 11 human BC samples from patients diagnosed with invasive BC undergoing mastectomy at Tongji Hospital, Tongji Medical College, Huazhong University of Science and Technology were collected for hematoxylin/eosin (H&E) and immunohistochemistry (IHC) staining. The Image J software (National Institute of Health, MD, USA) was used to measure the size of adipocytes in H&E-stained sections. The clinicopathological details of the 11 BC patients were displayed in Table S1.

### Cell culture and preparation of CAAs

4T1 cells and RAW 264.7 cells were collected from the Cell Bank of Chinese Academy of Sciences (Shanghai, China). 3T3-L1 preadipocytes, MDA-MB-231 and MCF-7 cells were purchased from Procell Life Science & Technology (Wuhan, China). Breast cancer cells and 3T3-L1 preadipocytes were cultivated in DMEM-H medium with 10% fetal bovine serum (FBS, Gibco, USA). RAW 264.7 cells were cultured in MEM-α medium with 10% fetal bovine serum. Through adipogenic differentiation of 3T3-L1 preadipocytes, mature adipocytes were obtained and identified by Oil Red O staining of the lipid droplets (Servicebio, Wuhan, China) [18]. Then, CAAs were acquired by co-culture of the mature adipocytes (upper chamber) with 4T1 cells (lower chamber) in a transwell co-culture system (pore size 0.4 μm, Corning, USA) for 3 days.

### Acquisition of conditioned medium

CAAs, mature adipocytes, and 4T1 cells were cultured in an FBS-free medium for 24 h to collect the CAA-conditioned medium (CM), AD-CM and 4T1-CM, respectively. Then, the collected CM was centrifuged at 3000 rpm for 10 min. To generate CAA-edu-RAW CM, AD-edu-RAW CM and 4T1-edu-RAW CM, RAW 264.7 cells were first cultured in CAA-CM, AD-CM and 4T1-CM for 72 h, respectively. Then, the CM was changed into FBS-free medium for 24 h. The FBS-free medium was collected as CAA-edu-RAW CM, AD-edu-RAW CM and 4T1-edu-RAW CM, respectively, with centrifugation at 3000 rpm for 10 min. 1% FBS was added to the CM in the following experiments.

### Proliferation and wound-healing assay

The cell counting kit (CCK-8; Yeasen, Shanghai, China) was used to assess cell proliferation according to the manufacturer’s instructions. Breast cancer cells were seeded at 5 × 10^3^/well in 96-well plates and cultivated in different CM. Then, each well was added with CCK-8 reagent at 0,12, 24 and 36 h. The enzyme-labeled instrument (Bio Tek, VT, USA) was utilized to test the optical density (OD) at 450 nm.

Breast cancer cells were inoculated at 1 × 10^6^/well in a 24-well plate and cultured to confluence. The10 μL pipette was used to scratch a straight line in the cells of each well. Then, cells were cultured with or without CM for 24 h. The Image J software was employed for analyze the wound healing condition of 5 different fields obtained by a microscope (SOPTOP CX40, Ningbo, China).

### Transwell migration and invasion assay

A total of 5 × 10^4^ 4T1 cells were cultivated in the upper chamber of the transwell system (pore size 8 μm, Corning, USA) in a 24-well plate for the transwell migration assay. The lower chamber of the transwell system was added with 500 μL CM. Then, the migrated 4T1 cells were dyed with crystal violet after incubation in the transwell system for 48 h. A microscope (SOPTOP CX40, Ningbo, China) was used to acquire the photographs of the migrated cells. The 33% glacial acetic acid was employed to wash the dyed cells. Finally, the OD values of each well were taken by the enzyme-labeled instrument at 590 nm.

As for the transwell invasion assay, the transwell chambers were coated with Matrigel (R&D system, MN, USA) of 3 mg/mL first. Then, a total of 2 × 10^5^ 4T1 cells were cultured in the upper chamber of the transwell system (pore size 8 μm, Corning, USA) in a 24-well plate. The protocols of the following steps were in accordance with the transwell migration assay.

### Quantitative real-time PCR (qRT-PCR)

TRIzol (Takara, Shiga, Japan) was applied to lyse the cultured cells. Total RNAs were extracted based on the manufacturer’s protocol of the 1st Strand cDNA Synthesis Kit (Yeasen, Shanghai, China) for subsequent cDNA synthesis. QuantStudio1 (ABI Q1, CA, USA) and SYBR GreenTM Master Mix (Yeasen, Shanghai, China) were employed for qRT-PCR. The main primer sequences applied for qRT-PCR were displayed in Table S2.

### Western blotting assay

IP lysis buffer with PMSF (Servicebio, Wuhan, China) was used to lyse cultured cells for western blotting assay. Then, protein quantification of the lysates was performed with the BCA Protein Assay Kit (Servicebio, Wuhan, China). Total proteins were first isolated on SDS-PAGE and the SDS-PAGE was cut according to protein molecular weight. And then, proteins were transferred onto the PVDF membranes (0.22 μm; Millipore, MA, USA). The PVDF membranes were blocked in 5% BSA and incubated at 4 °C overnight in specific primary antibodies. Subsequently, the membranes were incubated in HRP-conjugated secondary antibodies for 1 h at room temperature. Finally, ECL chemiluminescent reagents (Yeasen, Shanghai, China) were utilized to detect the protein bands. The original western blotting images were included in the Additional file 5 of the supplementary materials. The main antibodies applied in the western blotting assay were listed in Table S3.

### Animal models

BALB/C female mice of 6-8w purchased from Beijing Vital River Laboratory Animal Technology (Beijing, China) were involved in this study. All mice were bred at 21–25 ℃ and at 12/12 h of alternating light and dark. The animal experiments were performed with the approval of the Institutional Animal Care and Use Committee, Huazhong University of Science and Technology.

For constructing the subcutaneous tumor-bearing model, ten mice were randomly divided into two groups: the CAAs group and the control group. A total of 5 × 10^5^ 4T1 cells and 2.5 × 10^6^ adipocytes were injected into the axilla of the mouse subcutaneously in the CAAs group. A total of 5 × 10^5^ 4T1 cells were injected in the same location of the mouse in the control group. Mice were monitored and weighed every other day starting on day 1 after inoculation. The volume of the tumor was measured every 3 days when small and hard tumor nodules could be palpated. Four weeks after inoculation, mice were executed to obtain tumor tissues. Tumor tissues were weighed and were fixed in 4% paraformaldehyde and embedded in paraffin for following experiments.

For the tail vein pulmonary metastasis model, 4T1 cells were pre-cultured with CAA-CM or the control medium for 48 h and were injected into the tail vein of each mouse with 5 mice per group. After 14 days, all mice were sacrificed to isolate the lungs. Then, the lungs were embedded in paraffin for H&E staining to examine the pulmonary metastasis condition of each group.

### H&E, IHC and immunofluorescence (IF) assay

For the H&E staining assay, sample tissues were first fixed in 4% paraformaldehyde and were embedded in paraffin. Then, paraffin sections were cut and stained with hematoxylin and eosin. Representative photographs of H&E-stained sections were captured by the microscope (SOPTOP CX40, Ningbo, China).

For IHC assay, sections were first dissociated in xylene, and rehydrated in ethanol solutions with different concentrations. Sections were then heated in citrate buffer to recover the antigen and were blocked in 5% BSA. they were incubated with primary antibodies overnight at 4 °C, followed by HRP-conjugated secondary antibodies at room temperature for 30 min. Then the immunoreactivity of sections was visualized using DAB and counterstained with hematoxylin. Photographs of the sections were taken with the microscope (SOPTOP CX40, Ningbo, China).

For IF staining, the procedures prior to incubation of the secondary antibodies were consistent with IHC assay. Subsequently, sections were incubated in the fluorescently labeled secondary antibodies at room temperature for 1 h. The nuclei were then re-stained by nuclear 4,6-diamidino-2-phenylindole (DAPI). Photographs of the sections were taken by the fluorescence microscope (Olympus, Japan). The main antibodies used in the IHC and IF assay were involved in Table S3.

### Enzyme-linked immunosorbent assay (ELISA)

According to the manufacturer's introduction, mouse IL-6 ELISA kits (Invitrogen, CA, USA) were used to measure the secretion levels of IL-6 in mouse serum of CAAs group and the control group, and CAA-CM and the control medium.

### Statistical analyses

The statistical analyses of the experimental data were accomplished by Graphpad software (version 9.0, CA, United States). Data were obtained from at least three replicate assays and presented as mean ± standard deviation (means ± SD). Student’s-t test was used to compare the differences between two independent samples. One Way ANOVA was employed to analyze the differences among multiple groups.* P* < 0.05 was considered statistically significant.

## Results

### CAAs presented an alteration phenotype

**Fig. 1:**
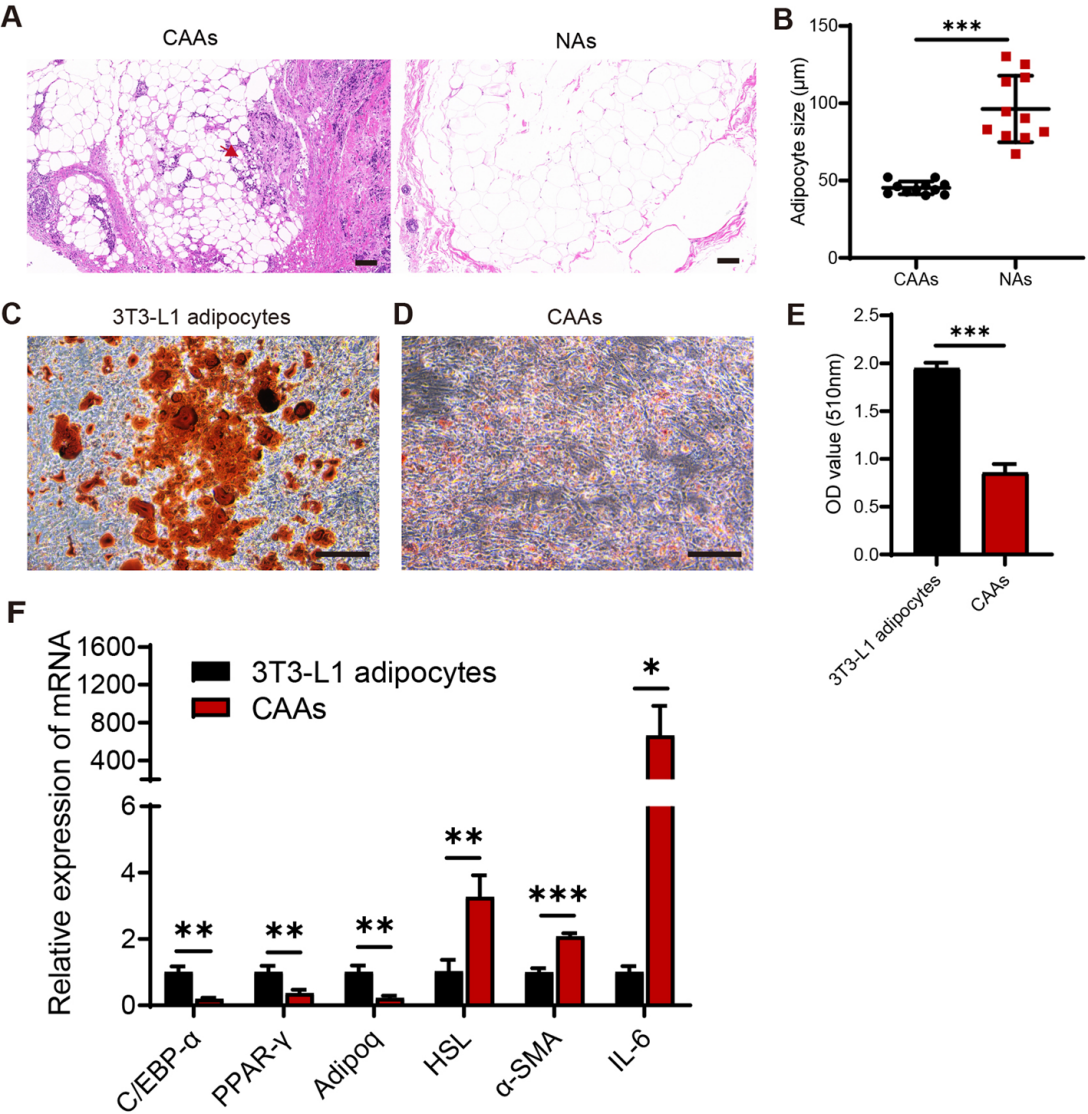
CAAs presented an alteration phenotype. **A** Representative images of H&E-stained adipocytes in human BC samples, including CAAs located at the invasive front of BC (red arrow) and normal mammary adipocytes, 100 × . **B** The cell diameters of CAAs and NAs in human BC samples were evaluated with the Image J software (*n* = 11). **C** Mature 3T3-L1 adipocytes were gained by the adipogenic-induced differentiation of 3T3-L1. The lipid droplets and cell morphology of mature 3T3-L1 adipocytes were observed by Oil red O staining. **D** CAAs were achieved by co-culture of mature 3T3-L1 adipocytes with 4T1 cells in a transwell system for 3 days. The lipid droplets and cell morphology of CAAs were checked by Oil red O staining. **E** The number of lipid contents was determined by extracting Oil red O with isopropanol and examining the optical density (OD) of Oil red O at 510 nm. **F** The mRNA expression levels of mature adipocyte-markers (C/EBP-α, PPAR-γ, and Adipoq), preadipocyte-markers (HSL and α-SMA), and pro-inflammatory IL-6 in 3T3-L1 adipocytes and CAAs detected by qRT-PCR. Scale bar, 100 μm. *P < 0.05, **P < 0.01, ***P < 0.001

Human BC specimens were collected to explore the morphological characteristics of CAAs. The results of the H&E staining indicated that CAAs were located at the invasive front of the tumor (Fig. 1A). It was apparent that CAAs were smaller in diameter compared to the normal mammary adipocytes (NAs) (Fig. 1A-B). Mature adipocytes were obtained by adipogenic induction of 3T3-L1 cells, and presented large and round lipid droplets, as confirmed by oil red o staining (Fig. 1C). To validate the de-differentiation features of CAAs, CAAs were acquired by co-culture of mature adipocytes with 4T1 cells in a transwell system. It was found that CAAs were decreased in size and were elongated in shape similar to fibroblasts with dispersed and small lipid droplets, compared to the mature 3T3-L1 adipocytes (Fig. 1C-E). In the analysis of adipocyte-specific gene expression, the expression levels of mature adipocyte markers C/EBP-α, PPAR-γ, and Adipoq mRNA were remarkably decreased, and preadipocyte markers HSL and α-SMA, and pro-inflammatory IL-6 were remarkably increased in CAAs, compared with the mature 3T3-L1 adipocytes (Fig. 1F). Taken together, these results suggested that mature adipocytes could be changed into CAAs in contact with adjacent BC cells and presented a de-differentiation phenotype, which accorded with the earlier observations [19–21].

### CAAs facilitated the malignant behaviors of breast cancer cells in vitro

**Fig. 2:**
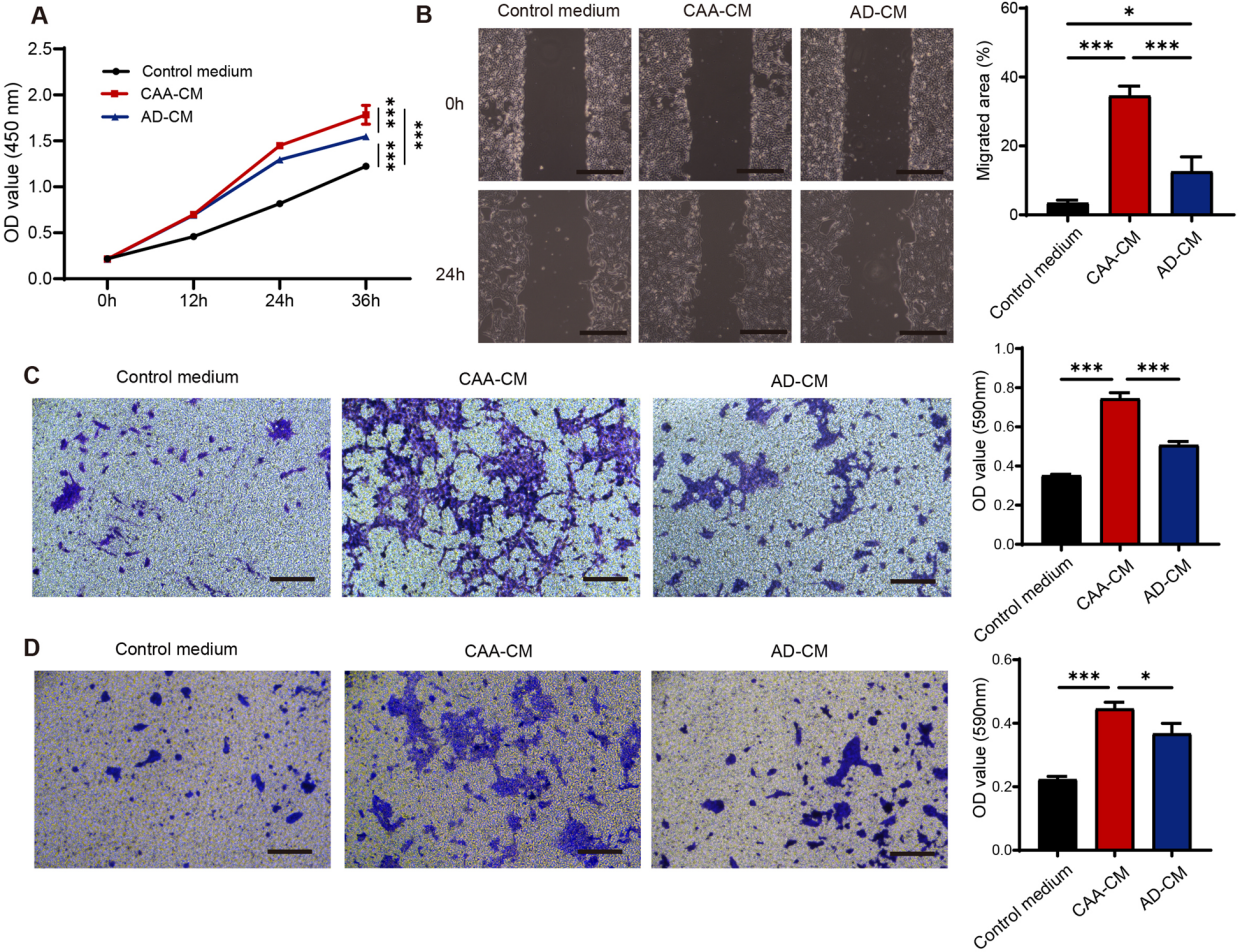
CAAs facilitated the malignant behaviors of breast cancer cells in vitro. **A** The proliferation ability of 4T1 cells assessed by CCK-8 assay cultivated in the control medium, CAA-CM or AD-CM, respectively. **B** The migration capability of 4T1 cells analyzed by wound healing assay cultured with the control medium, CAA-CM or AD-CM, respectively. **C** The migration capability of 4T1 cells was measured by the transwell migration assay treated with the control medium, CAA-CM or AD-CM, respectively, and the migrated cells were dyed in crystal violet and further quantified by checking OD values at 590 nm. **D** The invasion ability of 4T1 cells was examined by the transwell invasion assay in the above 3 groups, and the invaded cells were stained with crystal violet and further quantified by checking OD values at 590 nm. Scale bar, 100 μm. *P < 0.05, ***P < 0.001

Then, we examined the effect of CAAs on proliferation, migration and invasion of 4T1 cells. CAA-CM and AD-CM were obtained by culturing CAAs and mature adipocytes with serum-free DMEM-H medium, respectively, and were used to culture 4T1 cells. The results of CCK-8 assay showed that CAA-CM sharply increased the proliferation capability of 4T1 cells in comparison with AD-CM, and the pro-proliferation capability of AD-CM was also higher than the control medium (Fig. 2A). We also confirmed the significantly pro-proliferation effect of CAA-CM in MDA-MB-231 and MCF-7 human breast cancer cells when compared with the control medium (Fig. S1A-B). In addition, CAA-CM significantly promoted the migration ability of 4T1 cells, compared with AD-CM and the control medium, as was confirmed by the scratch assay and the transwell migration assay (Fig. 2B-C). In addition, CAA-CM notably enhanced the migration ability of MDA-MB-231 and MCF-7 cells in comparison with the control medium (Fig. S1C-D). Likewise, the invasion assay also indicated a pattern that CAA-CM efficiently facilitated the invasion ability of 4T1 cells (Fig. 2D). These data demonstrated that CAA-CM promoted the malignant behaviors of 4T1 cells.

### CAAs promoted the growth and metastasis of 4T1 cellsin vivo

**Fig. 3:**
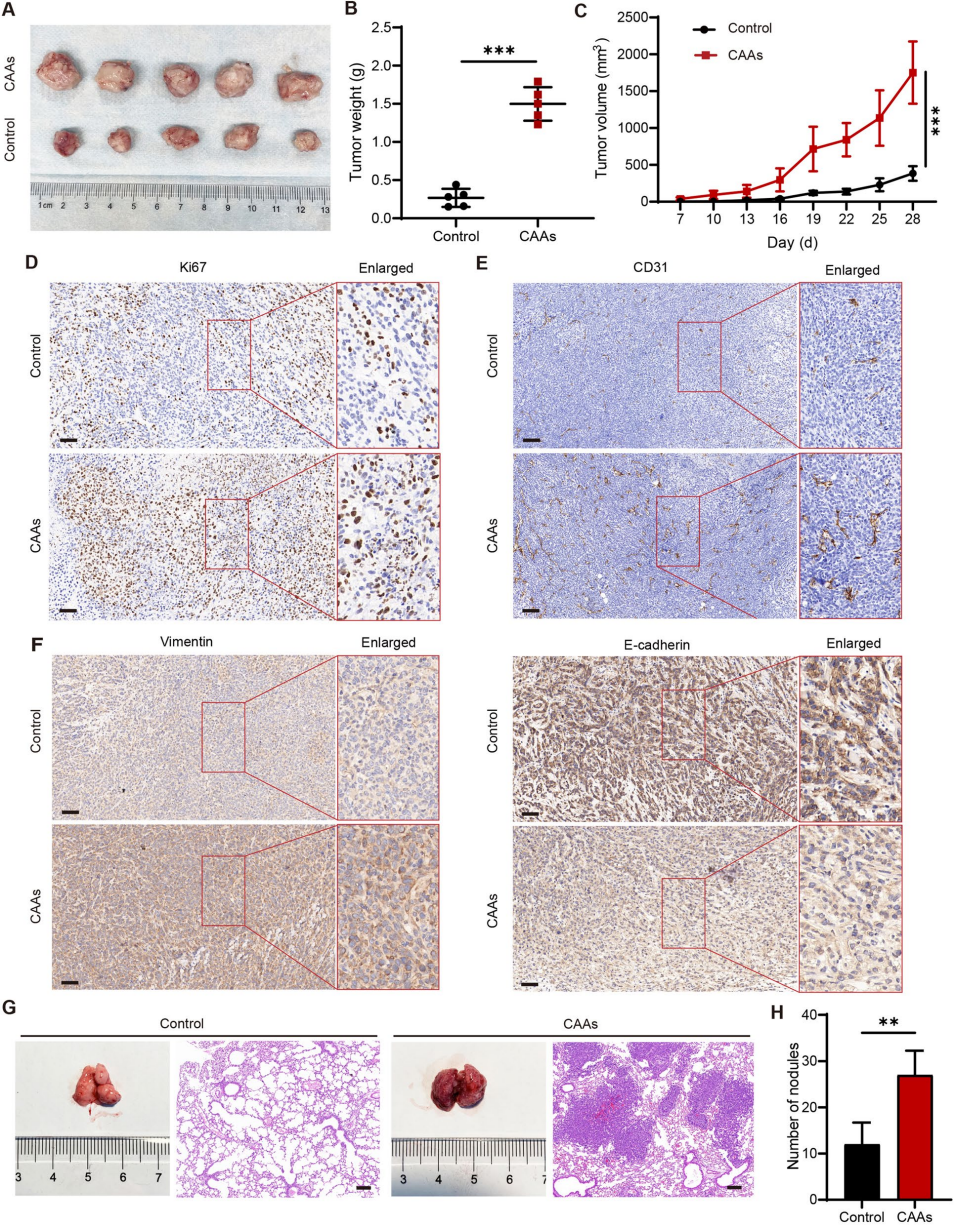
CAAs promoted the growth and metastasis of 4T1 cells in vivo. The mouse subcutaneous tumor-bearing model was established by co-injection of 4T1 cells with (CAAs group) or without (control group) mature 3T3-L1 adipocytes. **A** Tumors in the control group and the CAAs group acquired on day 28. **B** Tumor weight measured on day 28. **C** Tumor volume monitored every 3 days after palpating small and hard nodules. Representative IHC images of xenografts in the control group or the CAAs group with (**D**) Ki67, (**E**) CD31, (**F**) Vimentin and E-cadherin. The mouse tail vein pulmonary metastasis model was constructed by injecting CAA-CM pretreated 4T1 cells (CAAs group) or untreated 4T1 cells (control group) into the tail vein of the mouse. **G** Representative general and H&E images of the pulmonary metastasis in the control group or the CAAs group. **H** The number of pulmonary metastasis nodules in the control group or the CAAs group. Scale bar, 100 μm. **P < 0.01, ***P < 0.001

Subcutaneous tumor-bearing and tail vein pulmonary metastasis mouse models were constructed to validate the impact of CAAs on 4T1 cells in vivo. It was shown that the co-implanting of CAAs and 4T1 cells (CAAs group) significantly increased the volume and weight of 4T1 xenograft (Fig. 3A-B), in comparison with implanting 4T1 cells alone (control group), as well as the growth rate (Fig. 3C). The results of the IHC assay showed that the expression of cell proliferation marker Ki67 and endothelial cell marker CD31 of the xenograft were both more abundant in the CAAs group than in the control group, indicating a greater proliferative capacity and vascularization (Fig. 3D-E). The findings of the IHC assay also indicated that Vimentin expression was increased and E-cadherin expression was decreased in the CAAs group compared to the control group (Fig. 3F). Furthermore, the pulmonary metastasis model showed that CAA-CM-educated 4T1 cells accelerated the metastasis of 4T1 cells to the lung when compared with the 4T1 cells alone (Fig. 3G-H). These findings above indicated that CAAs could promote BC tumor growth, epithelial-to-mesenchymal transition (EMT), and pulmonary metastasis in vivo.

### CAAs enhanced infiltration of M2 macrophages in the TME of 4T1 BC

**Fig. 4:**
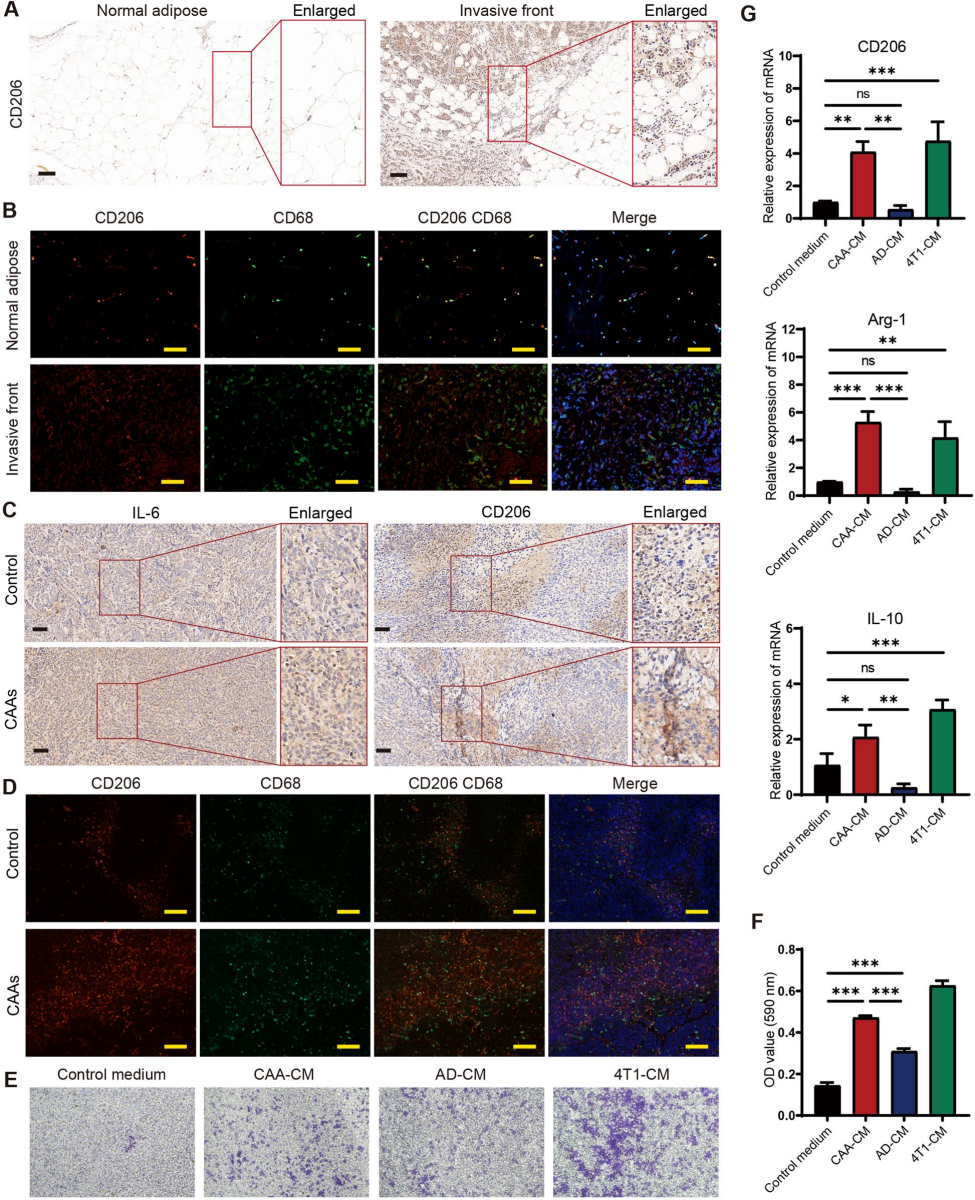
CAAs enhanced infiltration of M2 macrophages in the TME of 4T1 BC. **A** Representative IHC images of CD206 at the tumor invasive front compared to the normal breast adipose tissue. **B** Representative IF images presented the co-expression of CD206 and CD68 at the tumor invasive front compared to the normal breast adipose tissue. **C** Representative IHC images of IL-6 and CD206 in the xenografts of the control group or the CAAs group. **D** Representative IF images presented the co-expression of CD206 and CD68 in the xenografts of the control group or the CAAs group. **E** The migration capability of RAW 264.7 cells was measured by the transwell migration assay treated with the control medium, CAA-CM, AD-CM, and 4T1-CM, respectively, and (**F**) the migrated cells were stained with crystal violet and further quantified by checking OD values at 590 nm. **G** The mRNA expression levels of M2 macrophage markers (CD206, Arg-1, and IL-10) of RAW 264.7 cells treated with the control medium, CAA-CM, AD-CM or 4T1-CM, respectively detected by qRT-PCR. Scale bar, 100 μm. *P < 0.05, **P < 0.01, ***P < 0.001, ns: no significance

To further study the effect of CAAs on macrophages in the TME of 4T1 BC, we examined the expression of the M2 macrophage marker CD206 in human BC specimens, xenograft model and 4T1 cells. In human BC samples, CD206 expression tend to be more abundant at the invasive front of BC than in the normal adipose presented by IHC and IF assay, indicating more infiltration of M2 type macrophages at the invasive front of BC (Fig. 4A-B). The IHC assay further confirmed that the expression of CD206, as well as IL-6, was enhanced in the xenograft of CAAs group compared to the control group (Fig. 4C). The IF results showed more CD206 and CD68 co-localized M2 type macrophages in the xenograft of CAAs group (Fig. 4D).

Then, the transwell migration assay was performed to evaluate effect of CAAs, adipocytes and 4T1 cells on macrophages recruitment in vitro. The results demonstrated that both CAA-CM and 4T1-CM could effectively recruit macrophages, compared with AD-CM (Fig. 4E-F). It also presented that the expression of CD206, Arg-1 and IL-10, associated with M2 macrophages, was higher in CAA-CM educated macrophages than in AD-CM educated cells (Fig. 4G). Taken together, the results above suggested that CAAs might enhance the infiltration of M2 macrophages in the TME of 4T1 BC.

### CAA-educated macrophages promoted the malignant behaviors of 4T1 cells

**Fig. 5:**
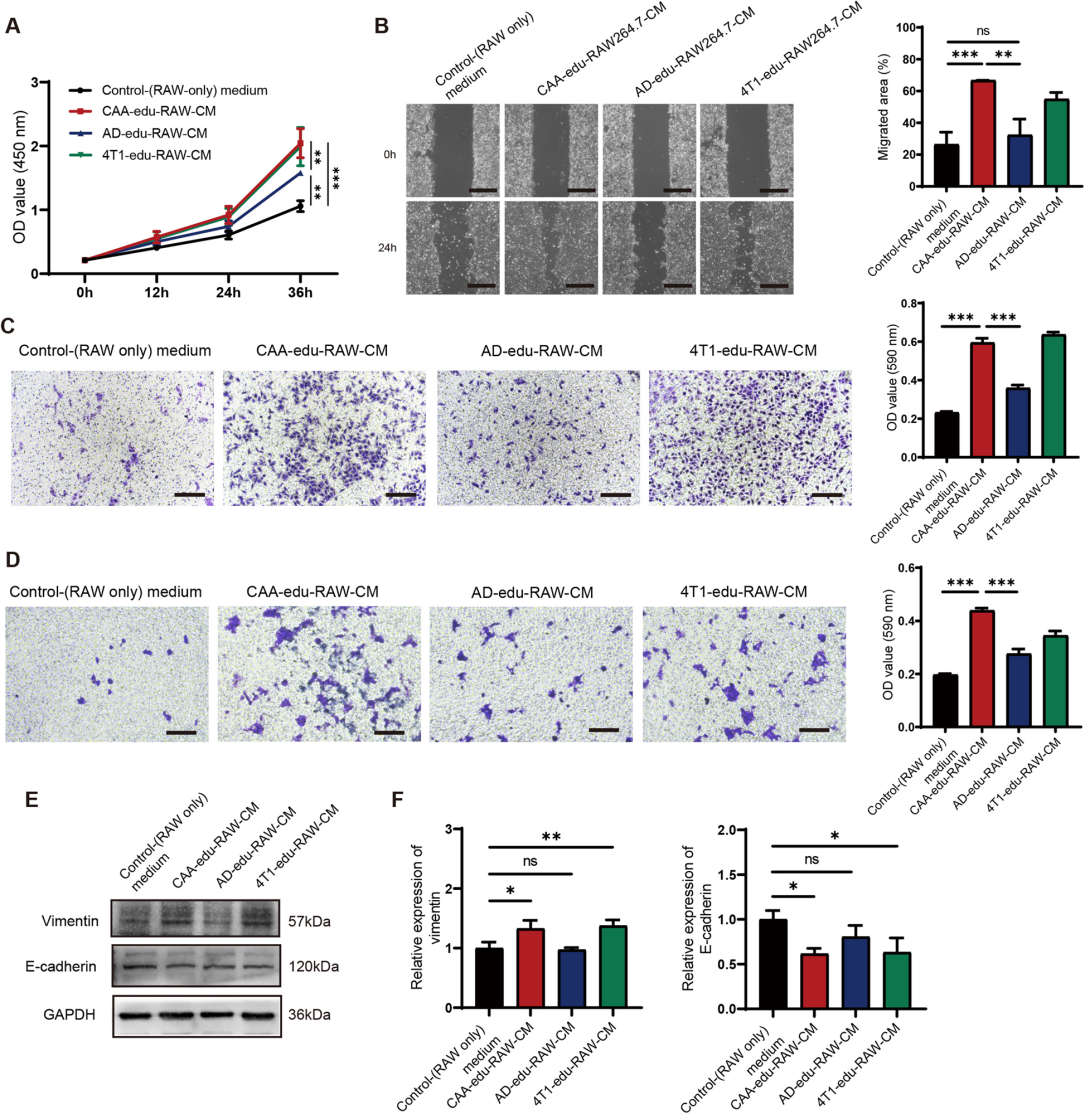
CAA-educated macrophages promoted the malignant behaviors of 4T1 cells. **A** The proliferation ability of 4T1 cells assessed by CCK-8 assay cultured in the control medium, CAA-edu-RAW-CM, AD-edu-RAW-CM, or 4T1-edu-RAW-CM respectively. **B** The migration capability of 4T1 cells analyzed by wound healing assay cultivated in the control medium, CAA-edu-RAW-CM, AD-edu-RAW-CM, or 4T1-edu-RAW-CM respectively. **C** The migration ability of 4T1 cells was measured by the transwell migration assay treated with the control medium, CAA-edu-RAW-CM, AD-edu-RAW-CM, or 4T1-edu-RAW-CM respectively, and the migrated cells were stained with crystal violet and quantified by examining OD values at 590 nm. **D** The invasion capability of 4T1 cells was examined by the transwell invasion assay in the above 4 groups, and the invaded cells were stained with crystal violet and further quantified by examining OD values at 590 nm. Scale bar, 100 μm. *P < 0.05, **P < 0.01, ***P < 0.001, ns: no significance

In order to explore the impacts of CAA-educated macrophages on malignant behaviors of BC cells, we treated 4T1 cells with CMs from CAA-, AD-, or 4T1- educated macrophages. The proliferation ability of 4T1 cells was enhanced by CAA-educated macrophages (similar to 4T1-educated macrophages), compared with AD-educated macrophages, and the pro-proliferation ability of AD-educated macrophages was higher than the control group (Fig. 5A). The scratch assay showed that CAA-educated macrophages significantly improved the migration ability of 4T1 cells (similar with 4T1-educated macrophages), whereas AD-educated macrophages did not significantly improve the migration ability, compared to the control macrophages (Fig. 5B). The transwell migration assay indicated that, compared with AD-educated macrophages, both CAA- and 4T1-educated macrophages significantly enhanced the migration ability of 4T1 cells, while the pro-migration ability of AD-educated macrophages was stronger than the control group (Fig. 5C). The invasion assay also confirmed a similar trend that CAA-educated macrophages effectively enhanced the invasion ability of 4T1 cells (Fig. 5D). Vimentin expression was increased and E-cadherin expression was decreased significantly in 4T1 cells cultured with CM from CAA-educated macrophages (similar to the 4T1-educated macrophages) than the control macrophages (Fig. 5E-F). These findings suggested that CAA-educated macrophages could promote the malignant behaviors of 4T1 cells.

### CAA-derived IL-6 induced M2 macrophage polarization by activating STAT3

**Fig. 6:**
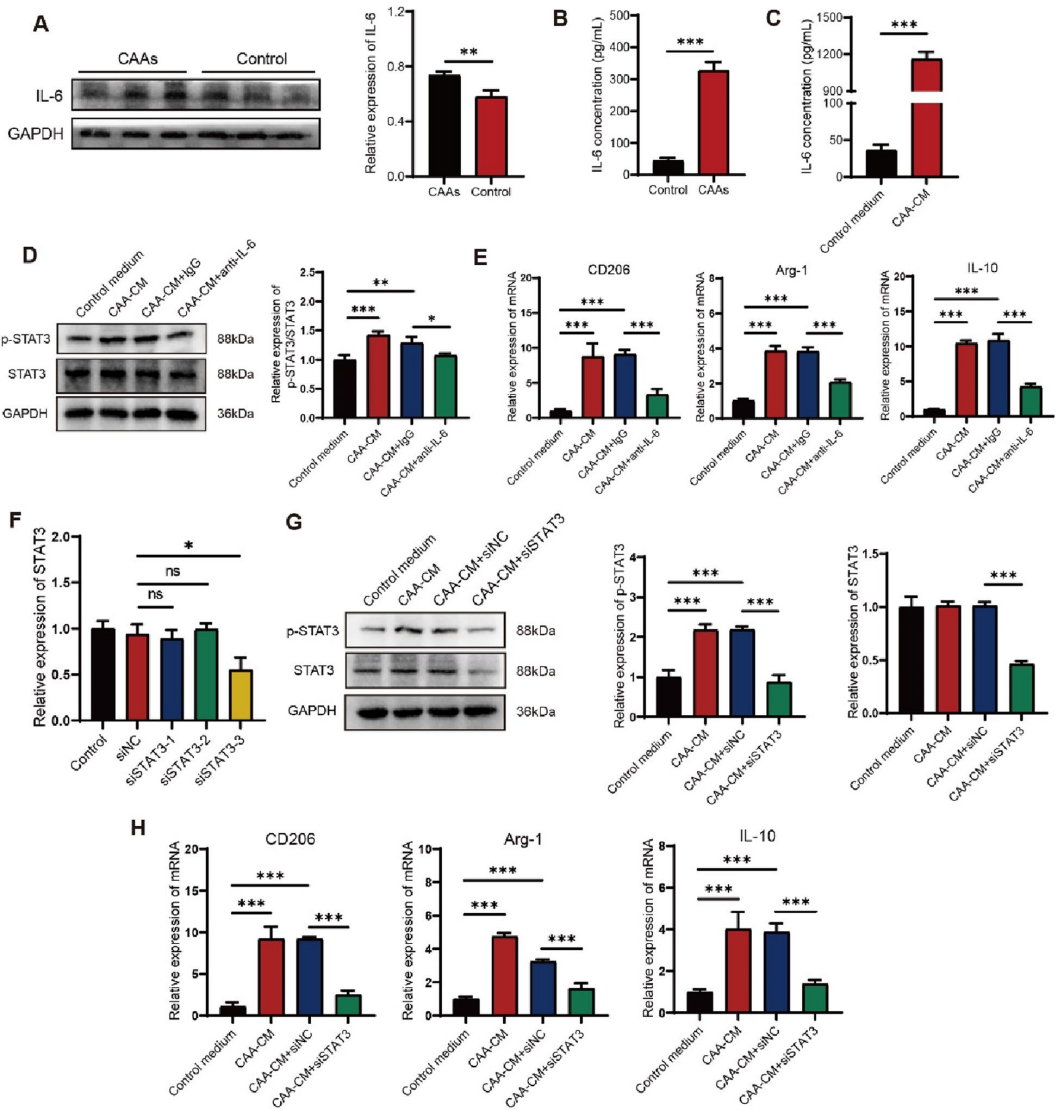
CAA-derived IL-6 induced M2 macrophage polarization by activating STAT3. **A** Western blotting assay was used to detect the in-situ IL-6 protein expression in the mouse tumor-bearing model in the CAAs group or the control group and quantified by Image J software and normalized to GAPDH. ELISA assay was employed to examine the IL-6 content (**B**) in serum of the mouse tumor-bearing model in the control group or the CAAs group and the IL-6 content (**C**) in the control medium or in the CAA-CM in vitro, respectively. **D** The protein expression of p-STAT3/STAT3 detected by western blotting assay in CAA-CM-educated RAW264.7 cells with the administration of an anti-IL-6 blocking antibody. **E** The mRNA expression levels of M2 macrophage markers (CD206, Arg-1, and IL-10) in CAA-CM-treated RAW 264.7 cells with the administration of an anti-IL-6 blocking antibody measured by qRT-PCR. **F** The knockdown efficiency of STAT3 assessed by qRT-PCR. **G** The protein expression of p-STAT3 and STAT3 detected by western blotting assay in CAA-CM-educated RAW264.7 cells transfected with si-STAT3. **H** The mRNA expression levels of M2 macrophage markers (CD206, Arg-1, and IL-10) in CAA-CM-treated RAW 264.7 cells transfected with si-STAT3. *P < 0.05, **P < 0.01, ***P < 0.001

To investigate the molecular mechanisms underlying macrophage polarization by CAAs, we focused on IL-6 which has been confirmed elevated significantly in the xenograft of CAAs group (Figs. 6A and 4C). The concentration of serum IL-6 detected by ELISA was higher in the CAAs group compared to the control group (Fig. 6B). The level of IL-6 was also higher in CAA-CM at the cellular level (Fig. 6C). The p-STAT3/STAT3 protein expression in RAW264.7 cells cultured with CAA-CM was significantly increased compared to the control medium (Fig. 6D). Inhibiting IL-6 activity through an anti-IL-6 blocking antibody (10 ng/mL) significantly reduced p-STAT3/STAT3 protein expression in RAW264.7 cells (Fig. 6D). The results of qRT-PCR assay showed that the expression CD206, Arg-1, and IL-10 was significantly decreased in the anti-IL-6 blocking antibody group compared to the isotype control group (Fig. 6E). The anti-IL-6 blocking antibody effectively reduced the pro-M2-macrophage ability of CAA-CM.

To further verify the role of STAT3 signaling in M2 macrophage polarization, siRNAs toward STAT3 were involved. si-STAT3-3 presented the best knockdown efficiency and was used in the following siRNA assay (Fig. 6F). The western blotting results showed that p-STAT3 and STAT3 protein expression was significantly decreased in si-STAT3-transfected RAW264.7 cells compared to si-NC-transfected RAW264.7 cells (Fig. 6G). Meanwhile, qRT-PCR results indicated that CD206, Arg-1 and IL-10 expressions were all significantly lower in si-STAT3-transfected RAW264.7 cells than in si-NC-transfected RAW264.7 cells (Fig. 6H). The above results suggested that CAA-derived IL-6 could promote M2 macrophage polarization via STAT3 signaling.

### CAAs upregulated PD-L1 expression in macrophages

**Fig. 7:**
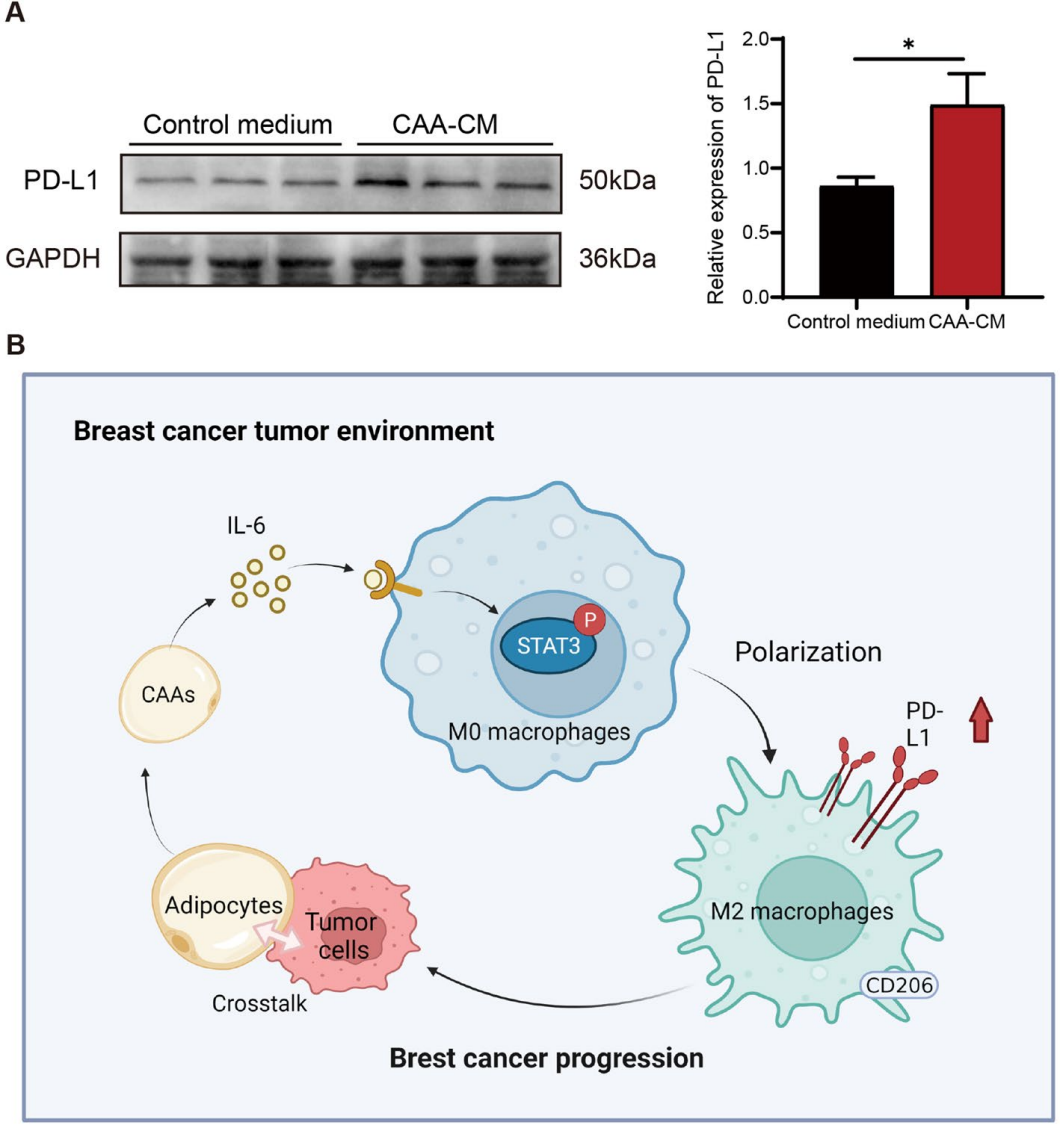
CAAs upregulated PD-L1 expression in macrophages. **A** The protein expression of PD-L1 examined by western blotting assay in CAA-CM-educated RAW264.7 cells compared with the control medium. **B** Schematic diagrams presented the mechanisms of CAAs in regulating macrophages and thus enhancing BC progression. *P < 0.05

The expression of programmed cell death protein ligand 1 (PD-L1) in CAA-CM-educated macrophages was detected by the western blotting assay, and the results showed that the PD-L1 expression was significantly higher in CAA-CM-educated macrophages than in the control cells (Fig. 7A). These results demonstrated that CAA-derived IL-6 could induce M2 macrophage polarization by activating STAT3 signaling, and promoted macrophage PD-L1 expression, thereby contributing to BC progression (Fig. 7B).

## Discussion

The continuous interaction between tumor cells and immune cells in the TME has an important role in tumor progression and metastasis. In addition, there are also interactions between the stromal cells and immune cells in the TME. In recent years, adipocytes at the invasive front of the tumor have been suggested to be associated with the recruitment and functional regulation of immune cells, thus influencing tumor cell behaviors [10]. In the present study, it was found that in human BC samples, adipocytes located at the invasive front of the tumor were smaller in size, and adipocytes underwent de-differentiated phenotypic changes into CAAs when co-cultured with BC cells. In vitro cellular assays revealed that CAA-CM could enhance the malignant behaviors of 4T1 cells. In the mouse model, CAAs could accelerate tumor growth and pulmonary metastasis. We also demonstrated that macrophages cultured with CAAs could further promote the malignant behaviors of 4T1 cells. Furthermore, we uncovered a potential mechanism that CAA-derived IL-6 could induce M2 macrophage polarization by activating STAT3 signaling.

CAAs have been first put forward by Diart et al. upon co-culture of murine 3T3-F442A adipocytes with tumor cells [22]. The research indicated that CAAs displayed a modified phenotype including a decrease in lipid content, a reduction in adipocyte markers (C/EBPα), and presented an activated condition with the characteristics such as overexpression of inflammatory cytokines (IL-6 and IL-1β) and proteases (matrix metalloproteinase-11) [22]. In turn, CAAs altered the features of tumor cells into a more aggressive phenotype [22]. Subsequently, Fujisaki et al. confirmed the findings of Dirat et al. in CAAs which were derived from BC patients, and further found that CAAs decreased in size and exhibited an immature and proliferative phenotype in the presence of cancer cells, and contributed to cancer cell migration via adipokines including IL- 6 and MCP-1 [23]. The morphometric study of adipocytes on BC verified that peritumoral adipocytes were smaller when compared with adipocytes of the normal tissues through an image analysis with photonic microscopy [24]. In our study, the phenotypic alteration of adipocytes with BC cells in vitro and in human BC samples were consistent with the characteristics of CAAs in the previous studies. Our study also indicated that CAAs could enhance the malignant behaviors BC cells both in vitro and in vivo. So far, studies on the interaction between CAAs and BC cells have mainly focused on the cellular level. In the present study, through co-injecting adipocytes and BC cells subcutaneously, we constructed a mouse tumor-bearing model and demonstrated that CAAs could promote breast tumor growth and malignant progression in vivo.

CAAs are important sources of adipokines, chemokines, cytokines, and exosomes that could promote tumor growth and progression [9]. Prior studies have noted the ability of CAAs in secreting high abundance of IL-6, IL-1β, CCL2, chemokine (C–C motif) ligand 5 (CCL5), tumor necrosis factor-α (TNF-α), vascular endothelial growth factor (VEGF) and leptin, which could enhance tumor invasion and immune escape [10, 22]. Increase secretion of IL-6 is one of the important phenotypic alterations of CAAs. Excessive secretion of IL-6 by tumor cells or mesenchymal cells in TME has been demonstrated to promote tumor growth, metastasis, and therapeutic resistance in multiple tumors, including BC [25, 26]. In the immune system, IL-6 has a dual effect: a pro-inflammatory or an anti-inflammatory factor, mainly depending on the local immune microenvironment [17, 27]. In some cases, IL-6 could induce M1 macrophage. It has been suggested that IL-6 induced macrophage expressed M1 macrophage marker, leading to an inflammatory TME thereby enhancing radiosensitivity in HPV^+^ head and neck cancer [28]. However, there is controversy regarding the role of IL-6 in inducing M1/M2 macrophage polarization. Braune et al. indicated that IL-6 in obesity adipose tissue acted as a Th2 cytokine by stimulating M2 macrophage polarization [29]. In addition, Weng et al. found that in triple-negative breast cancer (TNBC) cells, IL-6 stimulated by MCT-1 promoted monocytic THP-1 polarizing into M2 macrophages to enhance TNBC cell invasiveness in IL-6/IL-6R signaling [14]. The function of IL-6 in regulating macrophage polarization was complex and might be influenced by the local microenvironment.Here, we found that CAA-derived IL-6 could induce M2 macrophage polarization in vitro, which was in agreement with IL-6 involvement in M2 polarization in TNBC TME.

Macrophages are critical components of the host defense system [30]. So far, two polarization states of macrophages have been identified, including the pro-inflammatory classical activated (M1) macrophages and the anti-inflammatory alternative activated (M2) macrophages [31]. TAMs usually exhibited an M2 phenotype and promoted tumor progression by stimulating immunosuppression [31]. The activated state of TAMs was transient, leading to the functional plasticity of TAMs [1]. It is crucial to understand the role of macrophage heterogeneity and plasticity in the pro-tumor progression of CAAs. Adipocytes have been confirmed to affect macrophage polarization in adipose tissue and TME [32]. It has been recently reported that the uptake and oxidation of FFAs, released by abnormal catabolism of CAAs, were correlated with anti-inflammatory, immunosuppressive, and pro-tumor polarization of M2 macrophages [33]. CAA-secreted adenosine, accumulating in the tumor-associated adipose microenvironment, could reduce the classical polarization of macrophages or induce M2-type polarization and promote monocyte recruitment into tumors when it is binded to A2A or A2B receptors [11, 34]. In addition, lactate, the aerobic glycolytic end product of CAAs, regulated the polarization state of M2 macrophages through ERK/STAT3 signaling [12]. These results corroborated the findings of our study that CAAs were important participants in regulating M2 macrophage polarization. In this study, CAA-induced CD206 upregulation was found both in human samples and at the cellular level. However, the immunomodulatory function of CD206 has not been fully elucidated. The current study indicated that CD206 deficiency led to upregulation of the pro-inflammatory cytokine in serum mouse model, suggesting that CD206 might possess anti-inflammatory effects [35]. Based on this, CAA-induced increased expression of CD206 in macrophages with anti-inflammatory effects, which might be an indirect immunosuppressive mechanism of CAAs. It was reported that CD206 was more related to M2a macrophages, mediating TH2-type immune response [36]. Whereas, M2c macrophages, mainly expressed CD163, were more associated with the immune regulation and tissue remodeling [35, 37]. Therefore, further studies using CD163 as a marker are recommended to determine whether CAAs are more likely to induce immunosuppressation-associated M2c macrophages.

In TNBC, TAMs promote cancer growth and progression through multiple mechanisms and can directly and indirectly regulate PD-1/PD-L1 expression in TME [30]. The study of Zhang et al. indicated that in hepatocellular carcinoma, serum IL-6 could upregulate PD-L1 expression in macrophages, which in turn caused immunosuppression in an orthotopic tumor transplantation model [38]. Similarly, in the present study, we found that CAAs could upregulate PD-L1 expression in CAA-induced macrophages, and the upregulated PD-L1 might further enhance immunosuppression in TME of BC. There is abundant room for further progress in determining the complex mechanisms among CAAs, macrophages and BC cells. In addition to the soluble factor-mediated cell interaction in the present study, further research should be undertaken to investigate whether CAAs could directly induce immunosuppressive TME and inhibit T lymphocyte proliferation. In addition, it is necessary to study the correlation between the number of M2 macrophages and CAAs in human BC samples to determine whether CAAs and M2 macrophages coexist and thus assess the potential prognosis of BC.

## Conclusion

In general, this study identified that CAA-derived IL-6 could induce M2 macrophage polarization by activating STAT3 signaling, further supporting TNBC cell proliferation, invasion, metastasis and EMT. Moreover, CAAs could indirectly exert immunosuppressive function through upregulating PD-L1 in CAA-induced macrophages. The findings reported here provided a novel insight into the interaction and the potential mechanisms among CAAs, macrophages, and BC cells, shedding new light on anticancer strategies for BC therapy.

## Supplementary Information

**Additional file 1:** **Table S1.** Clinicopathological information of the 11 patients with breast cancer.

**Additional file 2:** **Table S2.** The primer sequences used for qRT-PCR.

**Additional file 3:** **Table S3.** Antibodies employed in the western blotting, IHC and IF assay.

**Additional file 4: Figure S1.** CAAs facilitated the malignant behaviors of human breast cancer cells in vitro.

**Additional file 5:** Uncropped western blot images.

## Data Availability

All data generated or analyzed during this study has been included in this article (and additional file).

## Abbreviations

TME: Tumor microenvironment
TAMs: Tumor-associated macrophages
BC: Breast cancer
IL: Interleukin
CAAs: Cancer-associated adipocytes
HGF: Hepatocyte growth factor
CCL2: Chemokine (C–C motif) ligand 2
H&E: Hematoxylin/eosin
IHC: Immunohistochemistry
CM: Conditioned medium
OD: Optical density
qRT-PCR: Quantitative real-time PCR
DAPI: Nuclear 4,6-diamidino-2-phenylindole
NAs: Normal mammary adipocytes
EMT: Epithelial-to-mesenchymal transition
PD-L1: Programmed cell death protein ligand 1
CCL5: Chemokine (C–C motif) ligand 5
TNF-α: Tumor necrosis factor-α
VEGF: Vascular endothelial growth factor
TNBC: Triple-negative breast cancer
