# Supplementary material for: CAA-derived IL-6 induced M2 macrophage polarization by activating STAT3
Source: BMC Cancer. 2023 May 1;23:392. doi: 10.1186/s12885-023-10826-1 (PMC10152707; doi:10.1186/s12885-023-10826-1)
Supplement: Supplementary file 1 — Additional file 1: Table S1. Clinicopathological information of the 11 patients with breast cancer. [file 12885_2023_10826_MOESM1_ESM.docx]

# Table S1. Clinicopathological information of the 11 patients with breast cancer

| Characteristics | Sub-groups | Number |
| --- | --- | --- |
| Age (years) | < 50 | 6 |
|  | ≥50 | 5 |
| Tumor size (cm) | <2 | 4 |
|  | ≥2 | 7 |
| TNM stage | I-II | 7 |
|  | III-IV | 4 |
| ER status | Negative | 4 |
|  | Positive | 7 |
| PR status | Negative | 5 |
|  | Positive | 6 |
| HER-2 status | Negative | 7 |
|  | Positive | 4 |
| Lymph nodal status | Negative | 6 |
|  | Positive | 5 |
