# Supplementary material for: CAA-derived IL-6 induced M2 macrophage polarization by activating STAT3
Source: BMC Cancer. 2023 May 1;23:392. doi: 10.1186/s12885-023-10826-1 (PMC10152707; doi:10.1186/s12885-023-10826-1)
Supplement: Supplementary file 2 — Additional file 2: Table S2. The primer sequences used for qRT-PCR. [file 12885_2023_10826_MOESM2_ESM.docx]

| mRNA | Species | Forward | Reverse |
| --- | --- | --- | --- |
| β-actin | Mouse | GGCTGTATTCCCCTCCATCG | CCAGTTGGTAACAATGCCATGT |
| C/EBP-α | Mouse | CAAGAACAGCAACGAGTACCG | GTCACTGGTCAACTCCAGCAC |
| PPAR-γ | Mouse | CTGTCAGAAACGGCTGTGTCA | TCCGGGCGAAACATTCA |
| Adipoq | Mouse | TGTTCCTCTTAATCCTGCCCA | CCAACCTGCACAAGTTCCCTT |
| HSL | Mouse | TCCCTCAGTATCTAGGCCAGA | GGCTCATTTGGGAGACTTTGTTT |
| α-SMA | Mouse | CCCAGACATCAGGGAGTAATGG | TCTATCGGATACTTCAGCGTCA |
| IL-6 | Mouse | GAGGATACCACTCCCAACAGACC | AAGTGCATCATCGTTGTTCATACA |
| STAT3 | Mouse | CACCTTGGATTGAGAGTCAAGAC | AGGAATCGGCTATATTGCTGGT |
| CD206 | Mouse | CTCTGTTCAGCTATTGGACGC | TGGCACTCCCAAACATAATTTGA |
| Arg-1 | Mouse | CTGGCAGTTGGAAGCATCTCT | GTGAGCATCCACCCAAATGAC |
| IL-10 | Mouse | CAGTACAGCCGGGAAGACAA | CCTGGGGCATCACTTCTACC |

# Table S2. The primer sequences used for qRT-PCR
