# Supplementary material for: CAA-derived IL-6 induced M2 macrophage polarization by activating STAT3
Source: BMC Cancer. 2023 May 1;23:392. doi: 10.1186/s12885-023-10826-1 (PMC10152707; doi:10.1186/s12885-023-10826-1)
Supplement: Supplementary file 3 — Additional file 3: Table S3. Antibodies employed in the western blotting, IHC and IF assay. [file 12885_2023_10826_MOESM3_ESM.docx]

# Table S3. Antibodies employed in the western blotting, IHC and IF assay

| Antibody | Species | Company |
| --- | --- | --- |
| anti-GAPDH | Mouse | Proteintech |
| anti-Vimentin | Mouse | Abclonal |
| anti-E-cadherin | Mouse | Abclonal |
| anti-Ki67 | Mouse | Proteintech |
| anti-CD31 | Mouse | Proteintech |
| anti-PD-L1 | Mouse | Proteintech |
| anti-IL-6 | Mouse | Proteintech |
| anti-CD206 | Mouse | Proteintech |
| anti-CD68 | Mouse | Proteintech |
| anti-p-STAT3 | Mouse | Proteintech |
| anti-STAT3 | Mouse | Proteintech |
| anti-CD206 | Human | Proteintech |
| anti-CD68 | Human | Proteintech |
| goat anti-mouse IgG | Goat | Proteintech |
| goat anti-rabbit IgG | Goat | Proteintech |
