# Supplementary figures and images for: CAA-derived IL-6 induced M2 macrophage polarization by activating STAT3
Source: BMC Cancer. 2023 May 1;23:392. doi: 10.1186/s12885-023-10826-1 (PMC10152707; doi:10.1186/s12885-023-10826-1)

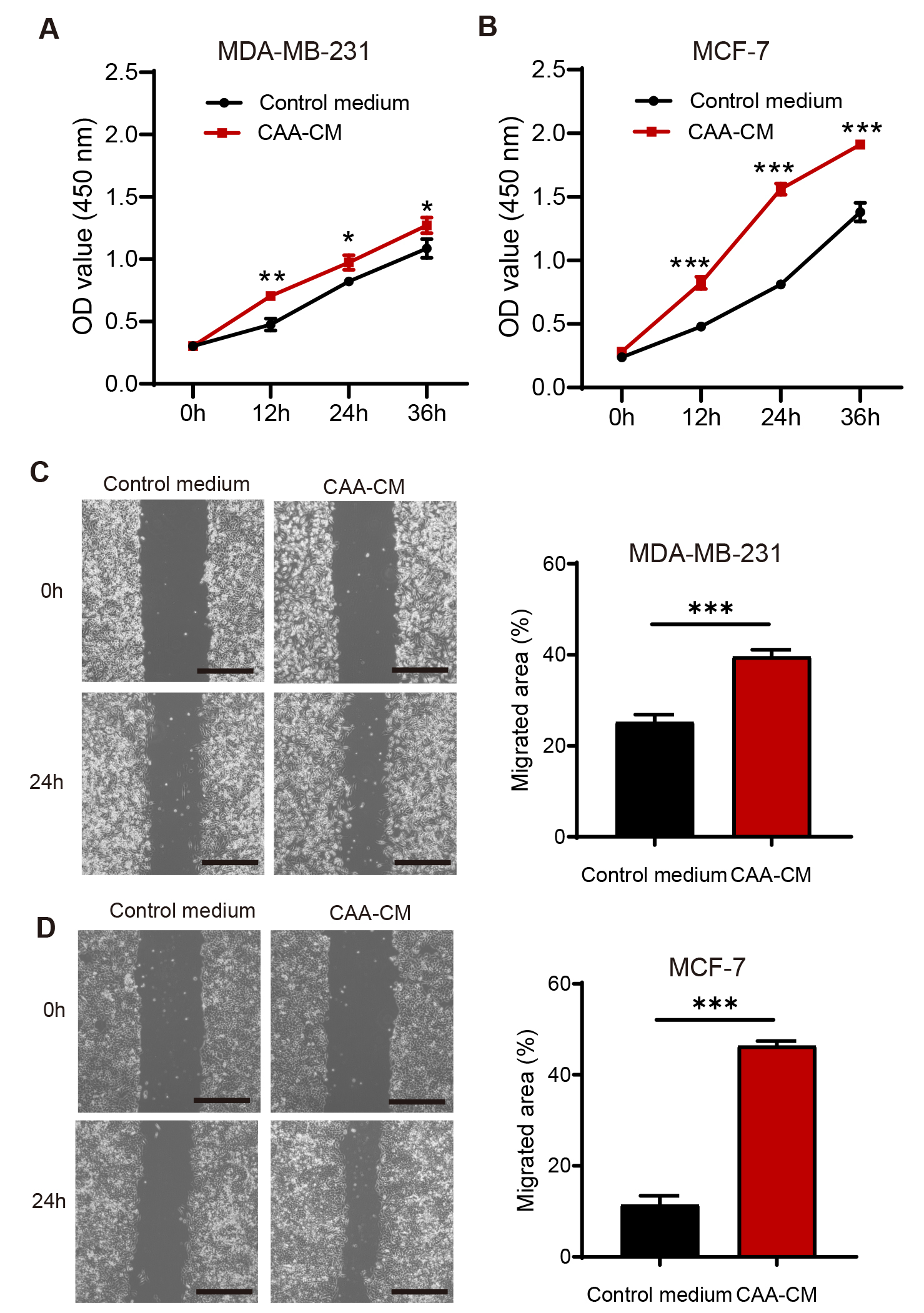

Supplement: Supplementary file 4 — Additional file 4: Figure S1. CAAs facilitated the malignant behaviors of human breast cancer cells in vitro. [file 12885_2023_10826_MOESM4_ESM.jpg]
