# Supplementary material for: CAA-derived IL-6 induced M2 macrophage polarization by activating STAT3
Source: BMC Cancer. 2023 May 1;23:392. doi: 10.1186/s12885-023-10826-1 (PMC10152707; doi:10.1186/s12885-023-10826-1)
Supplement: Supplementary file 5 — Additional file 5: Uncropped western blot images. [file 12885_2023_10826_MOESM5_ESM.docx]

**Uncropped western blot images**

**Figure 5E**

Vimentin


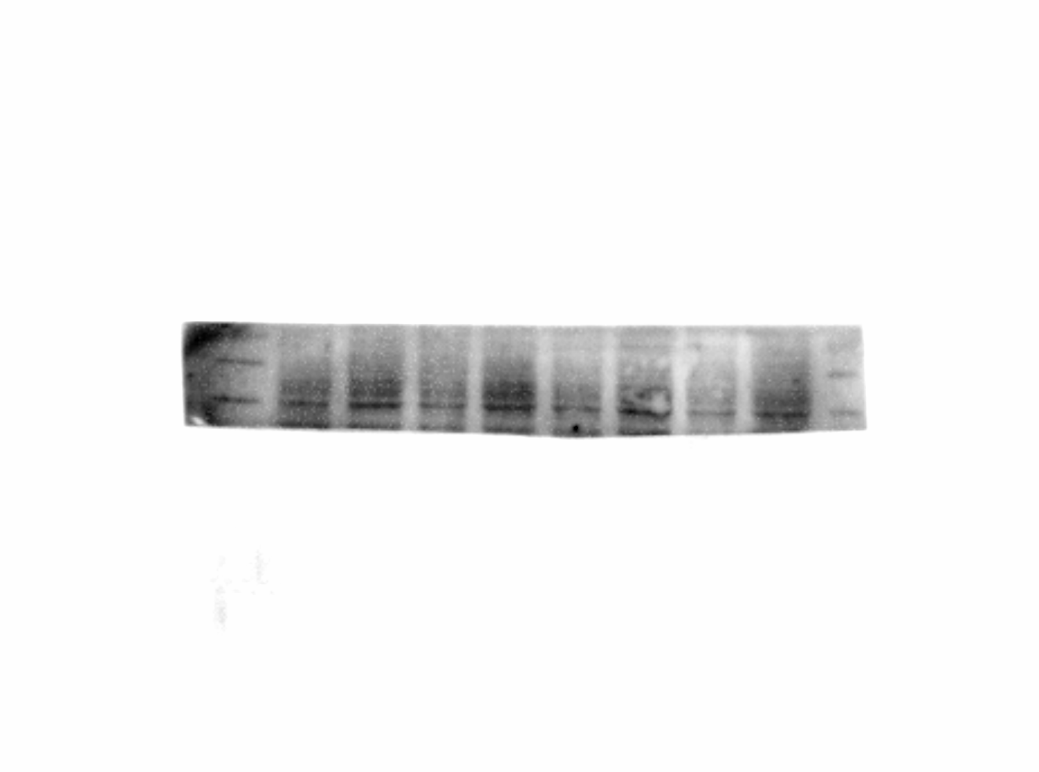


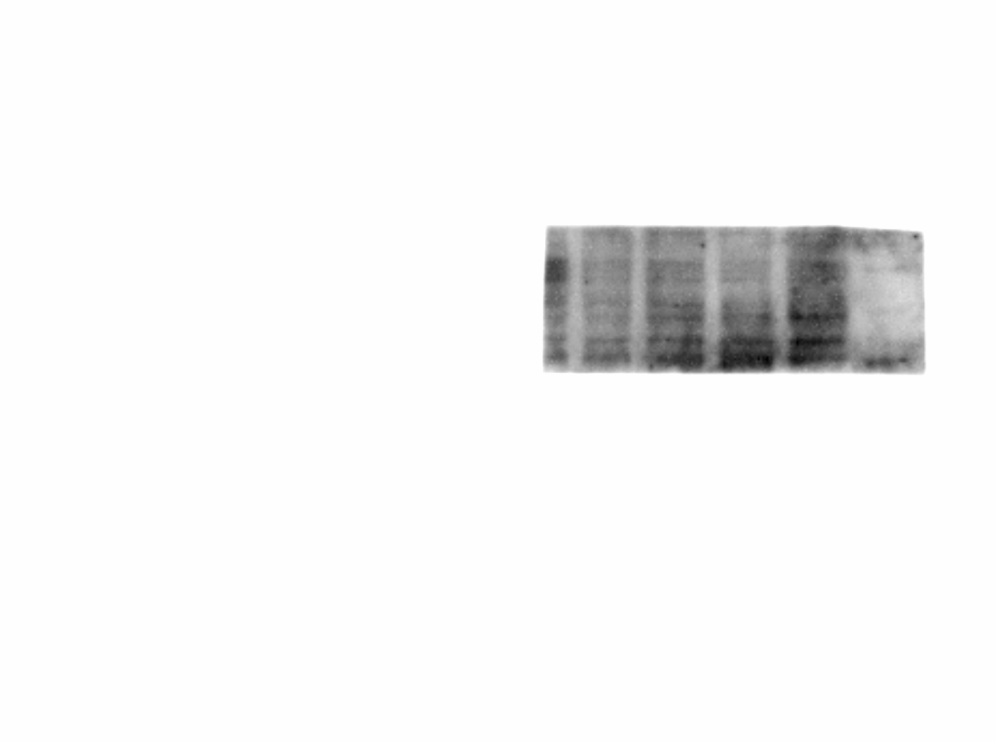


E-cadherin


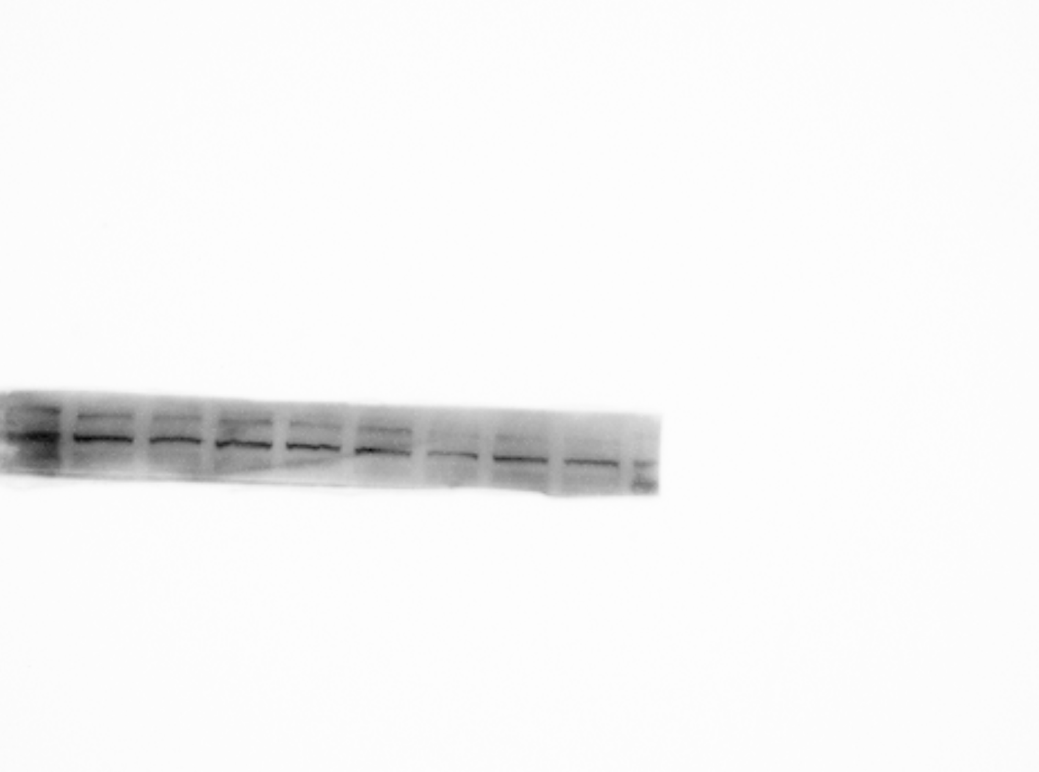


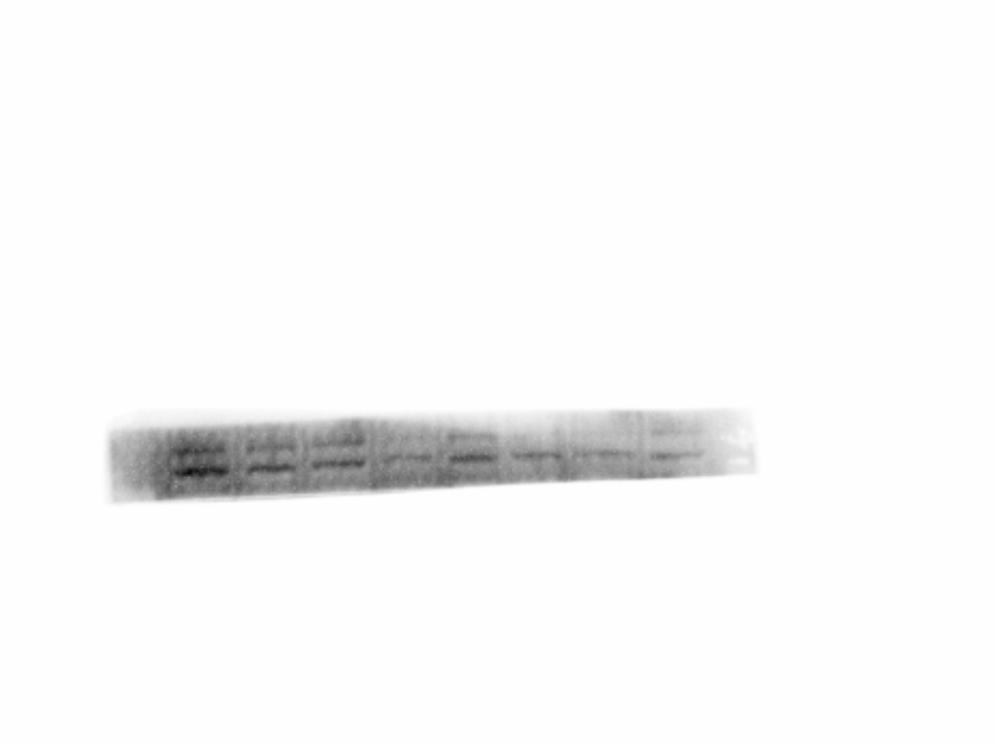
(the left 4 bands)

GAPDH


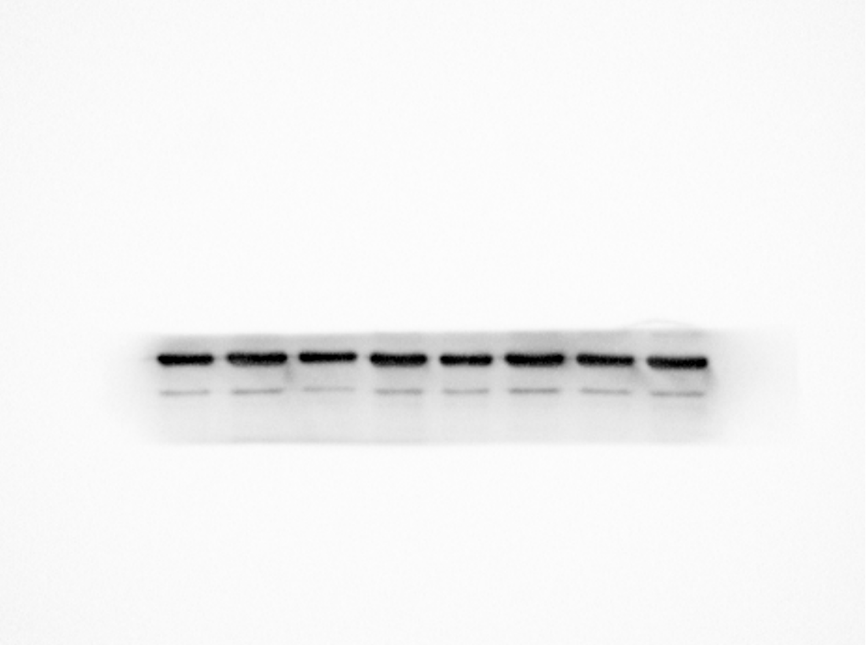

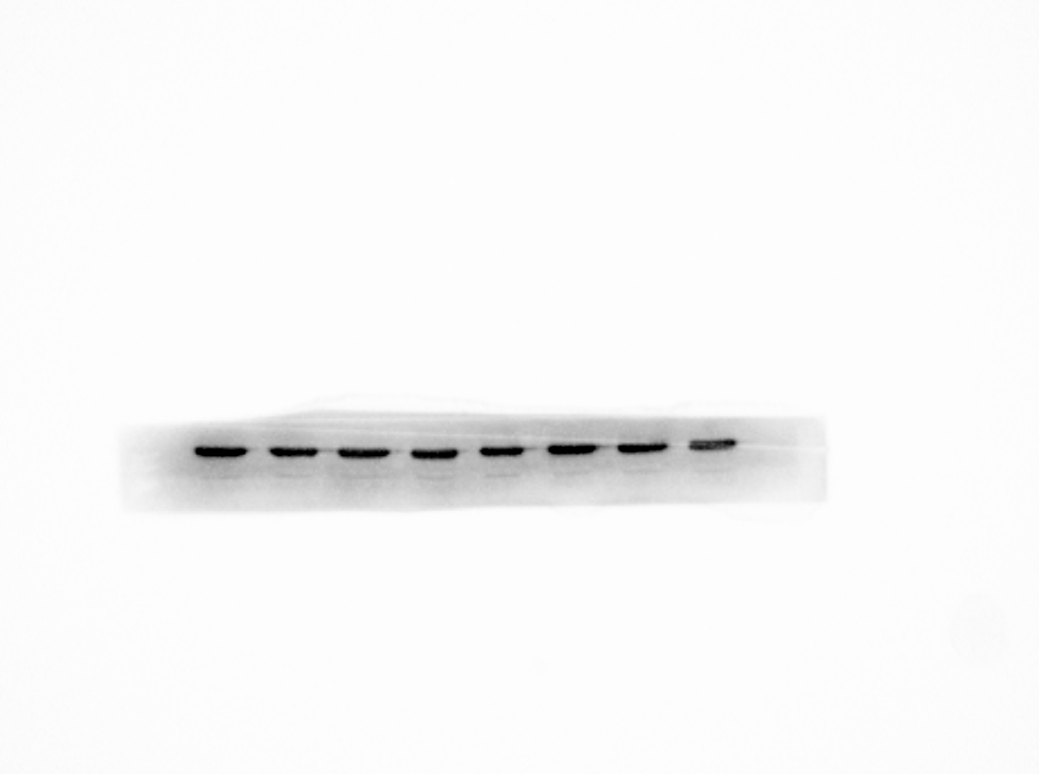


**Figure 6A**

IL-6


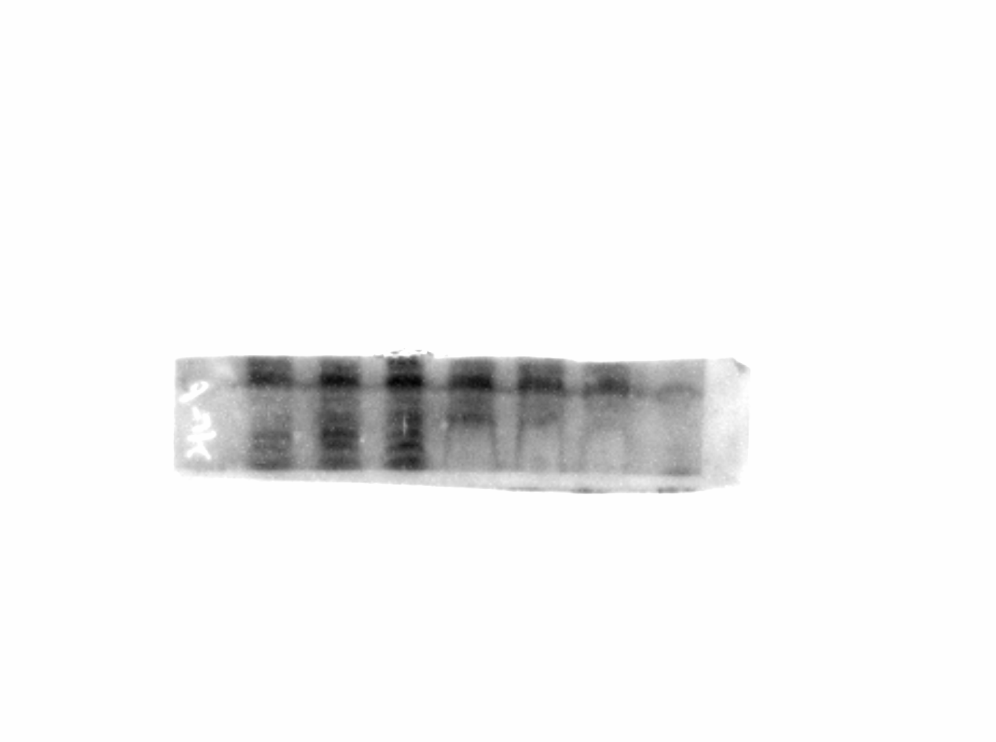


GAPDH


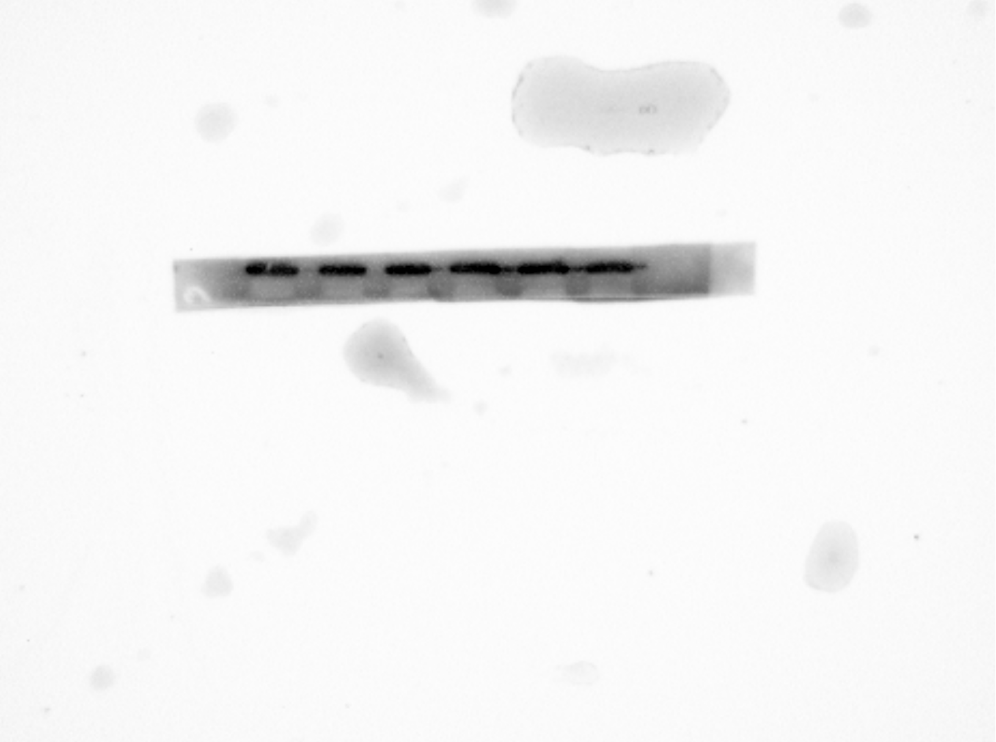


**Figure 6D**

p-STAT3


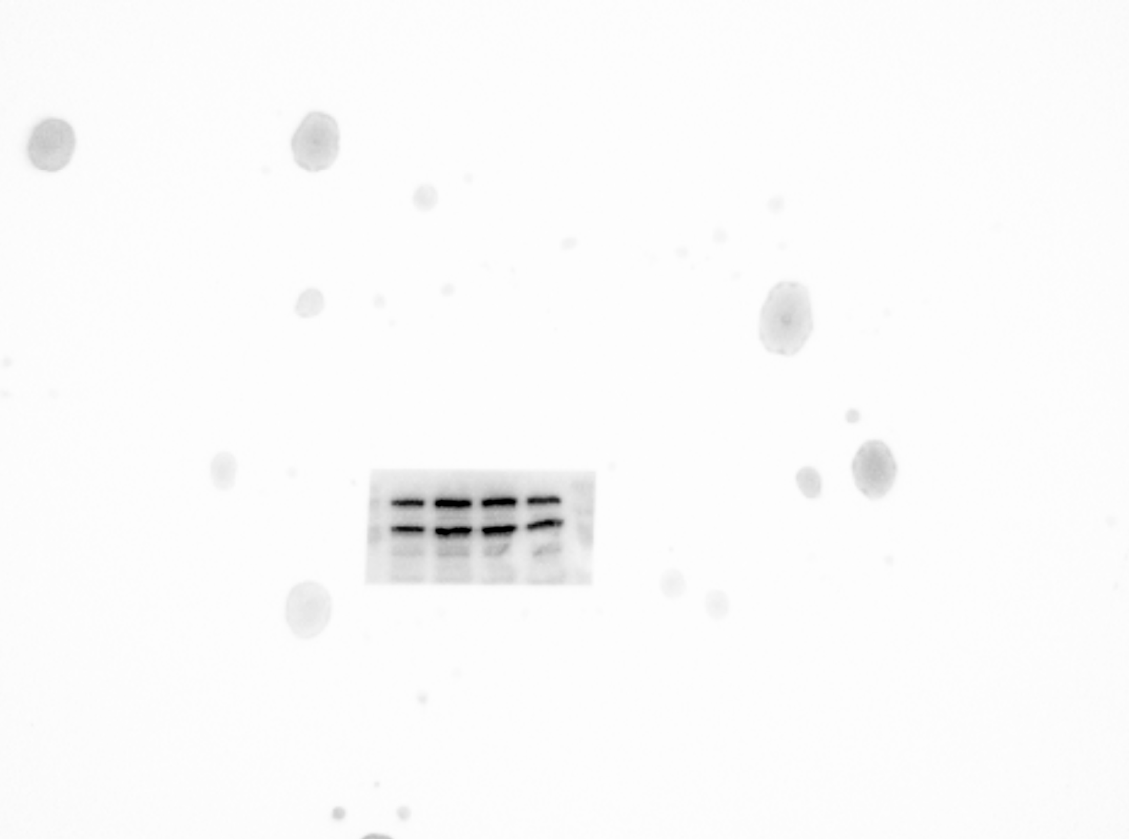


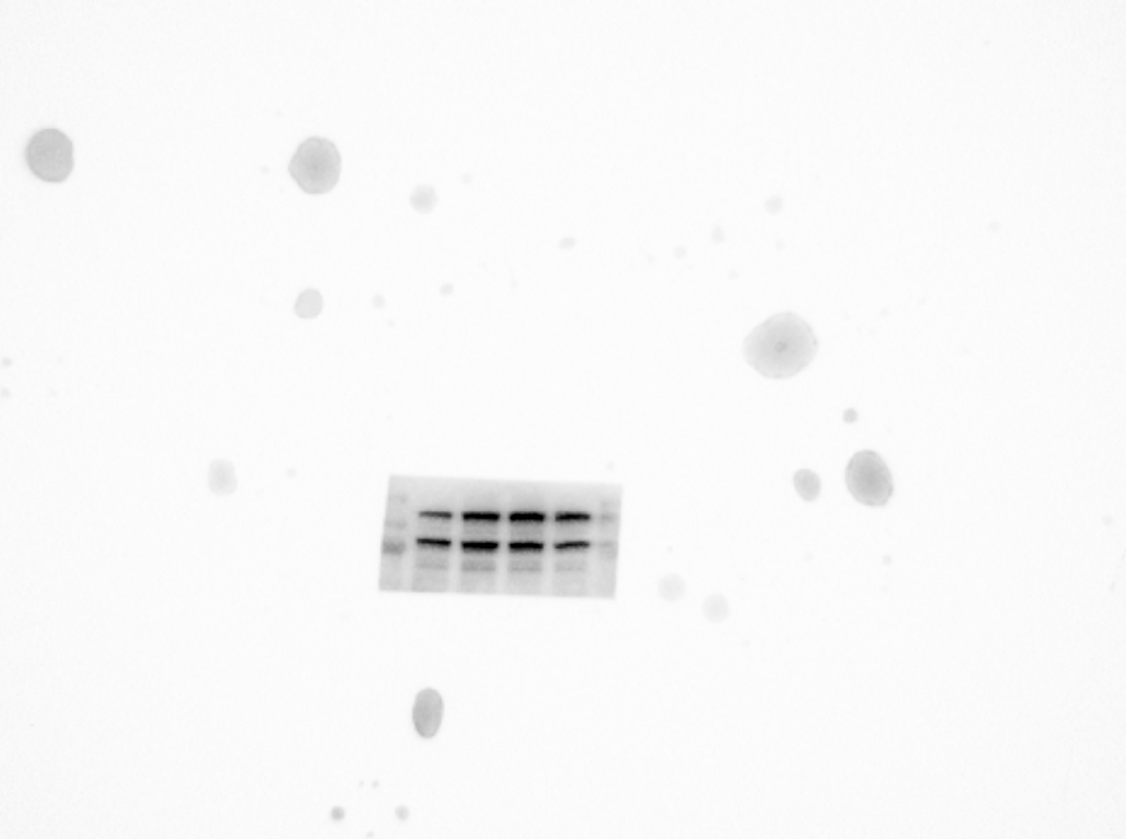


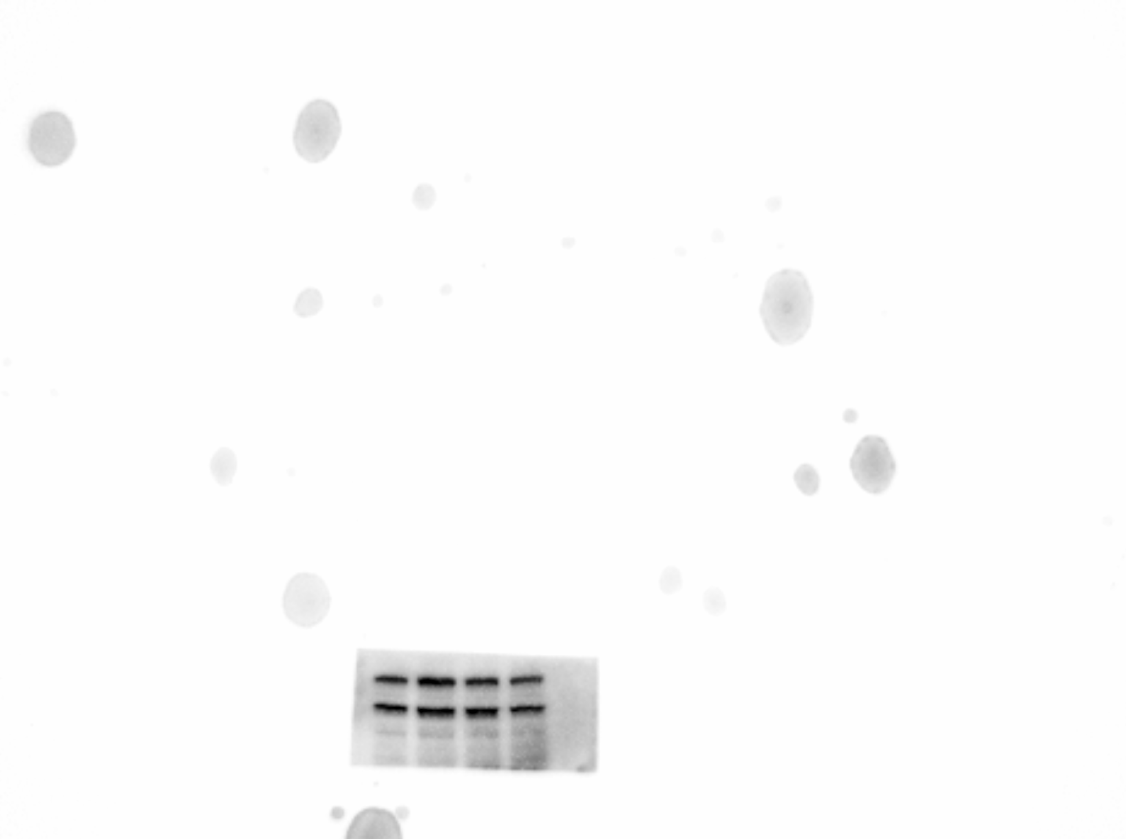


STAT3


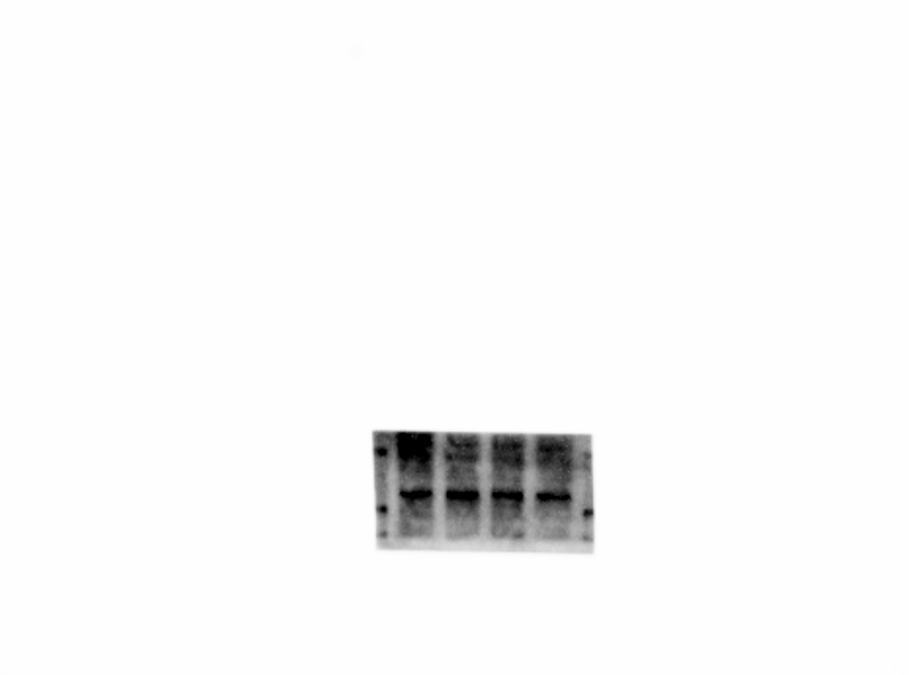


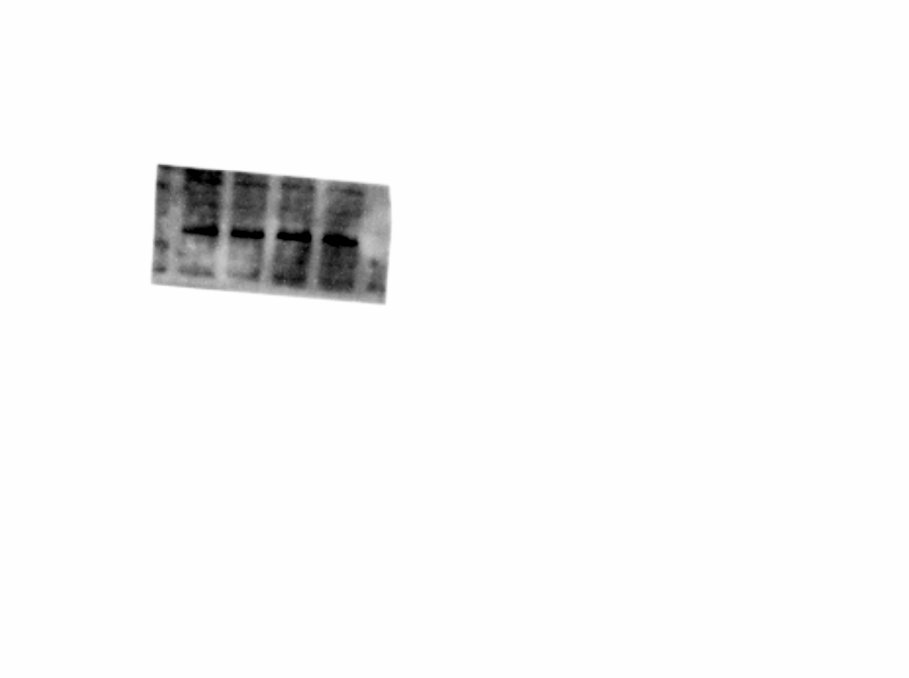


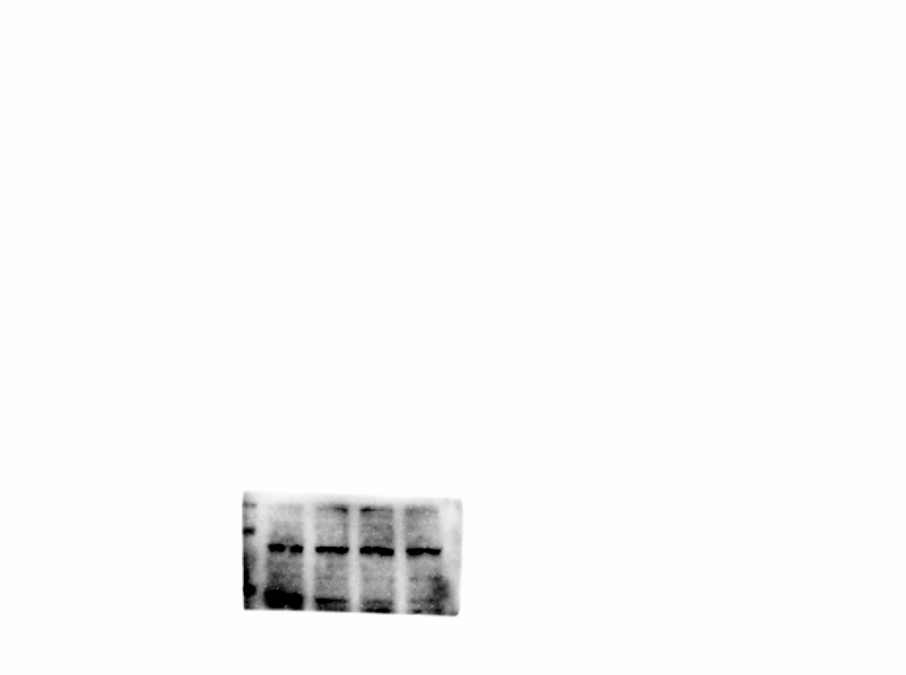


GAPDH


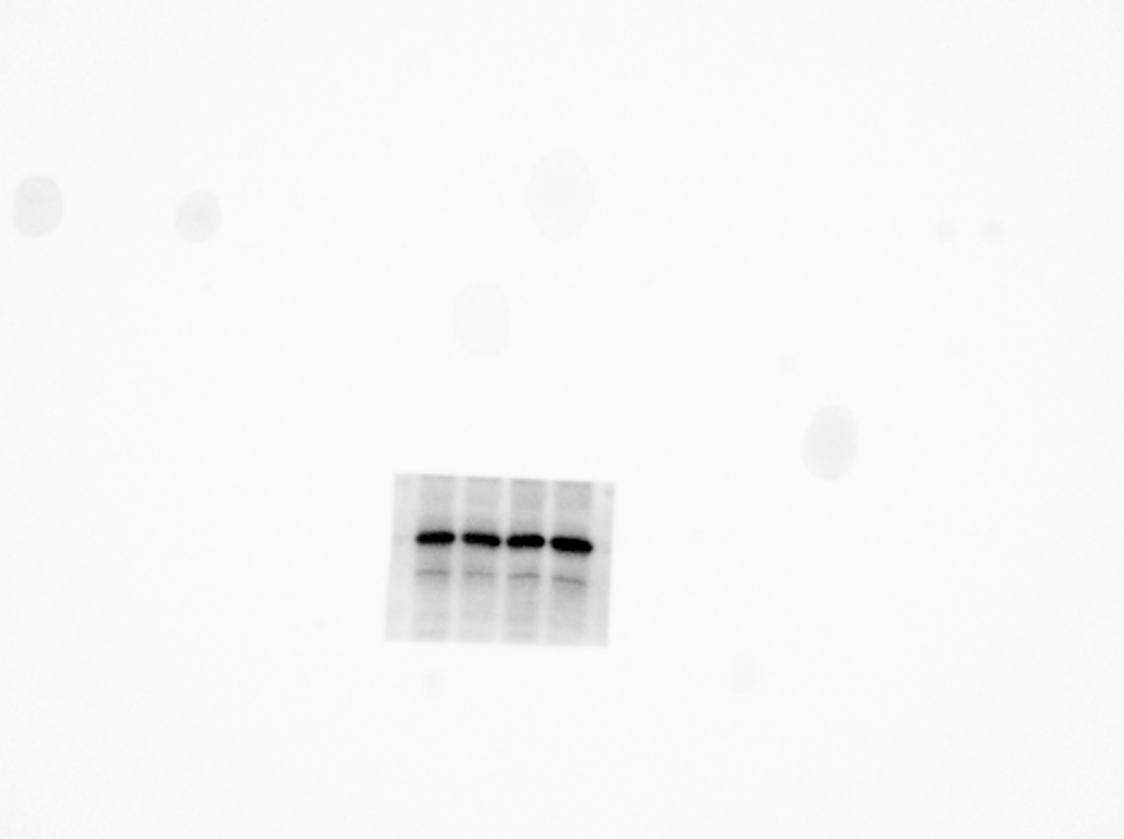


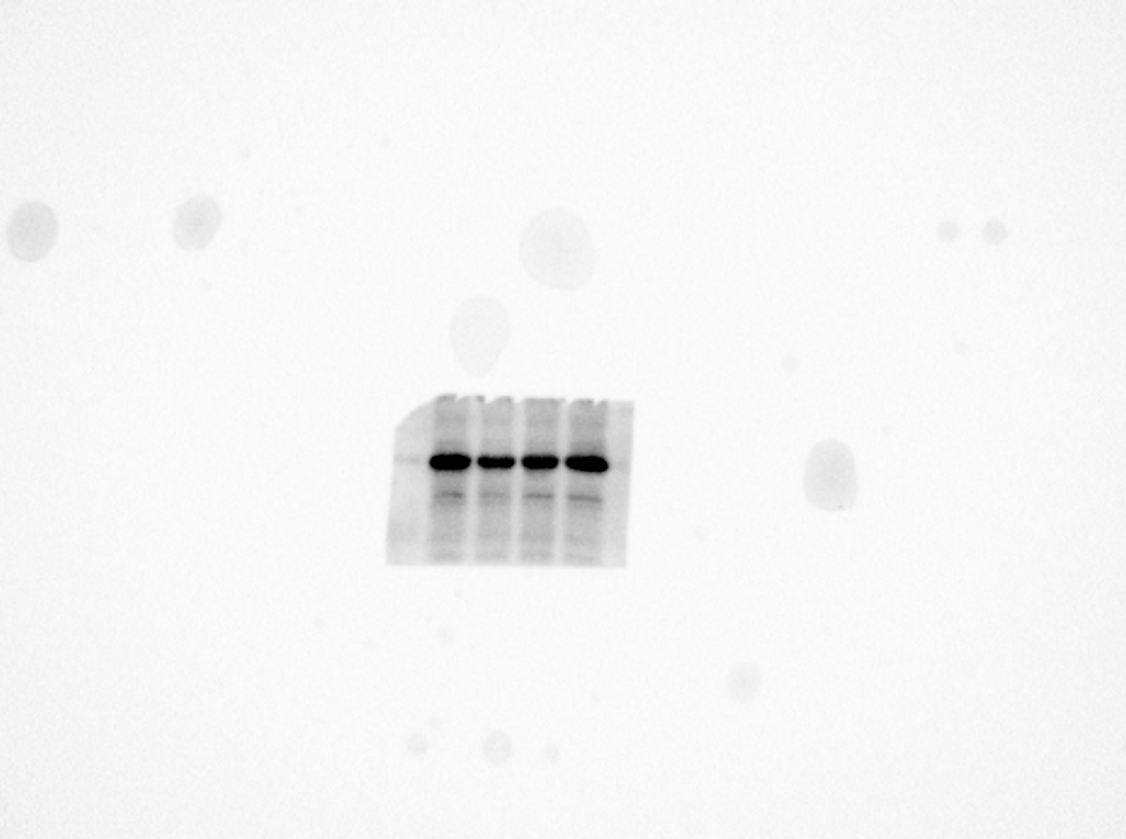


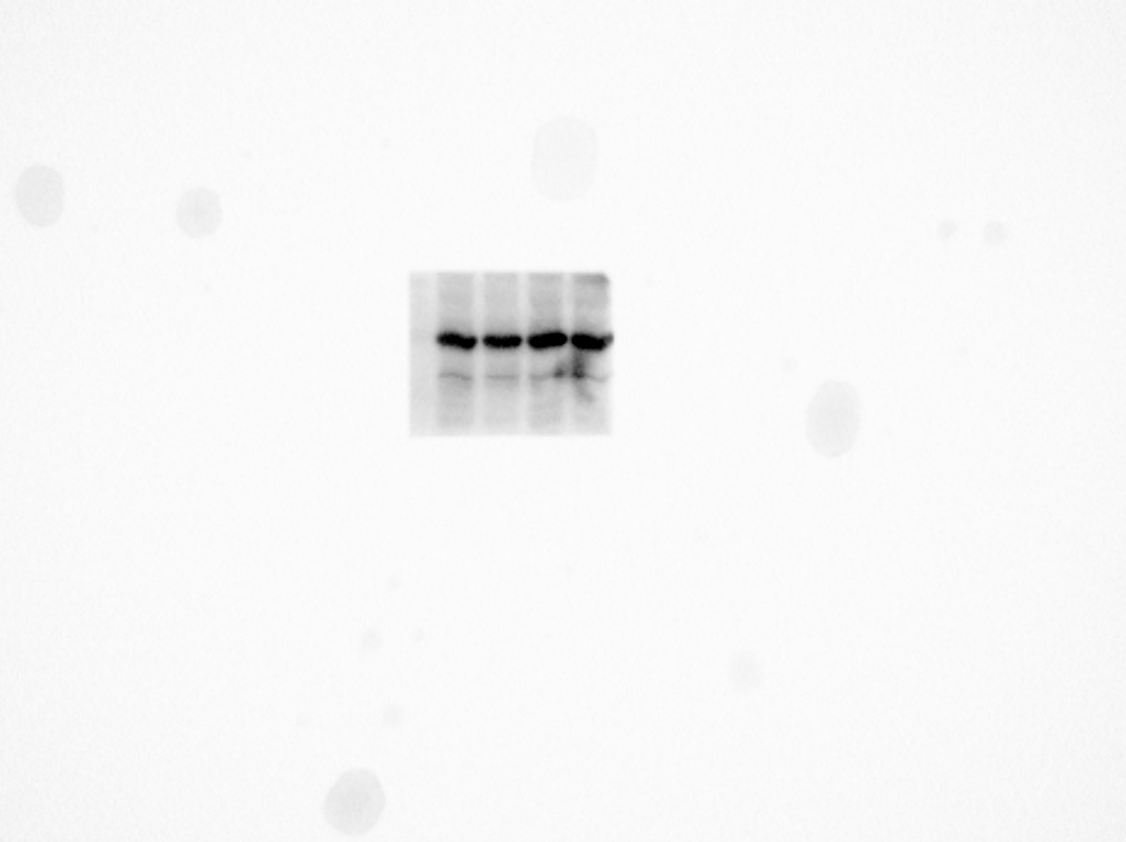


**Figure 6G**

p-STAT3


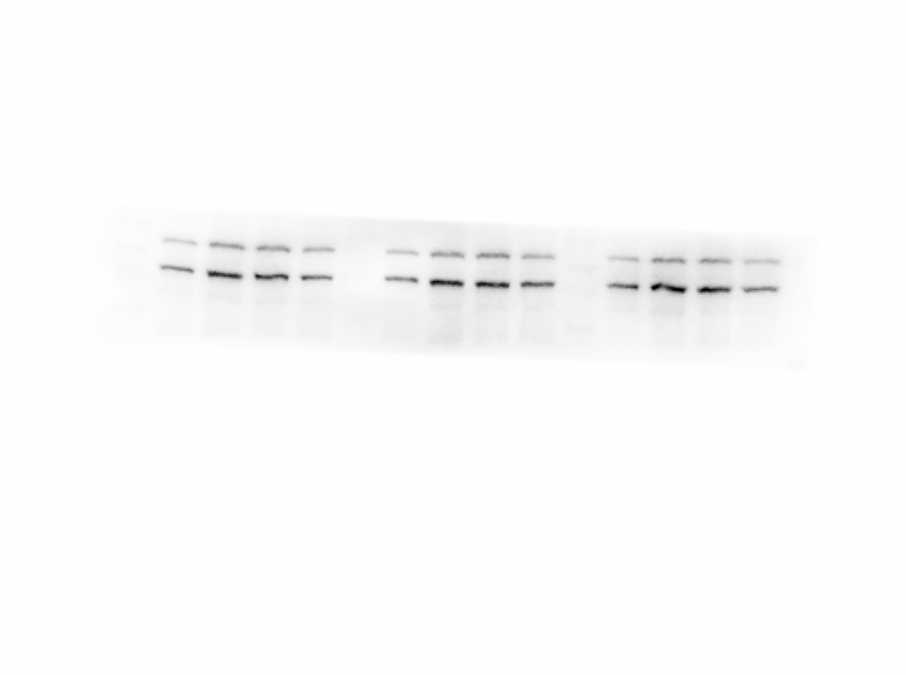


STAT3


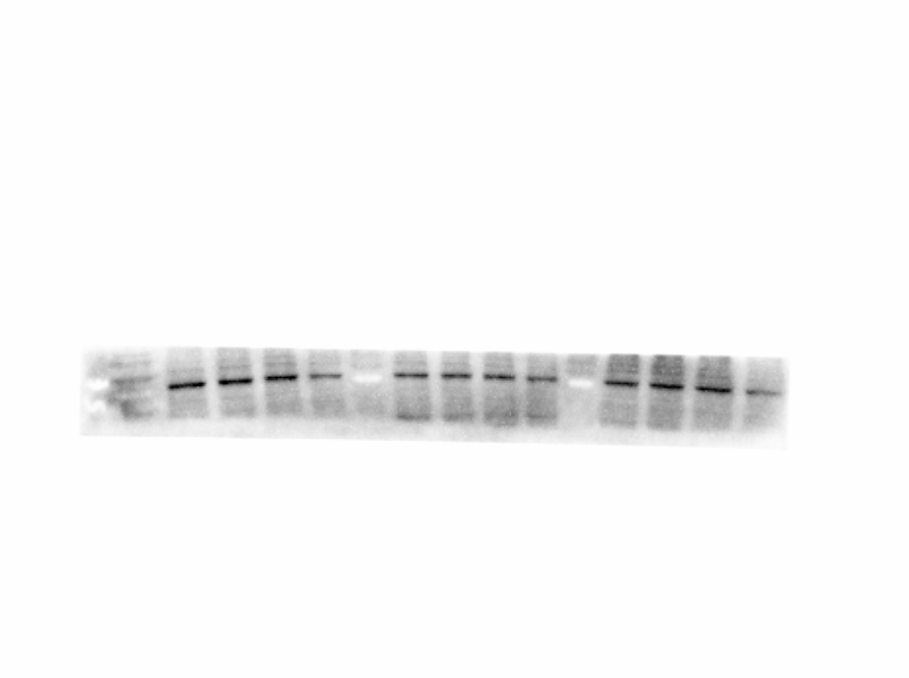


GAPDH


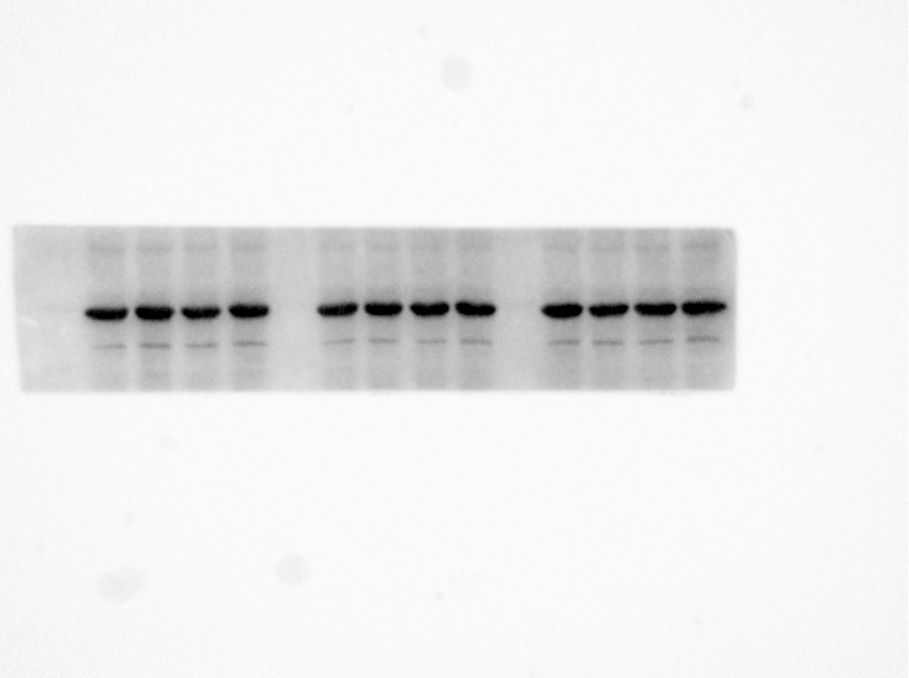


**Figure 7A**

PD-L1


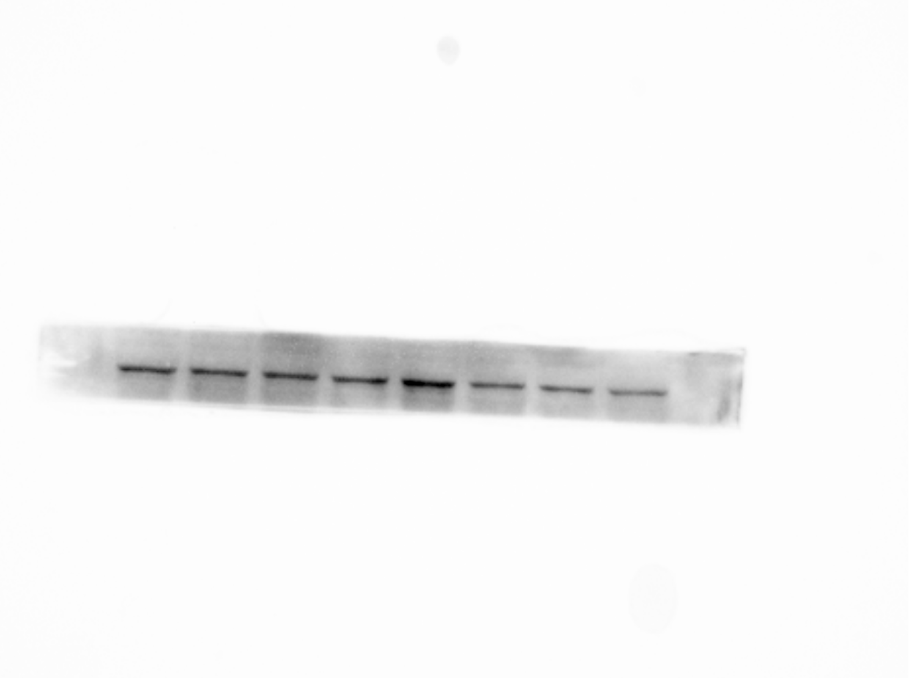


**CAA-CM**

**Control medium**

GAPDH


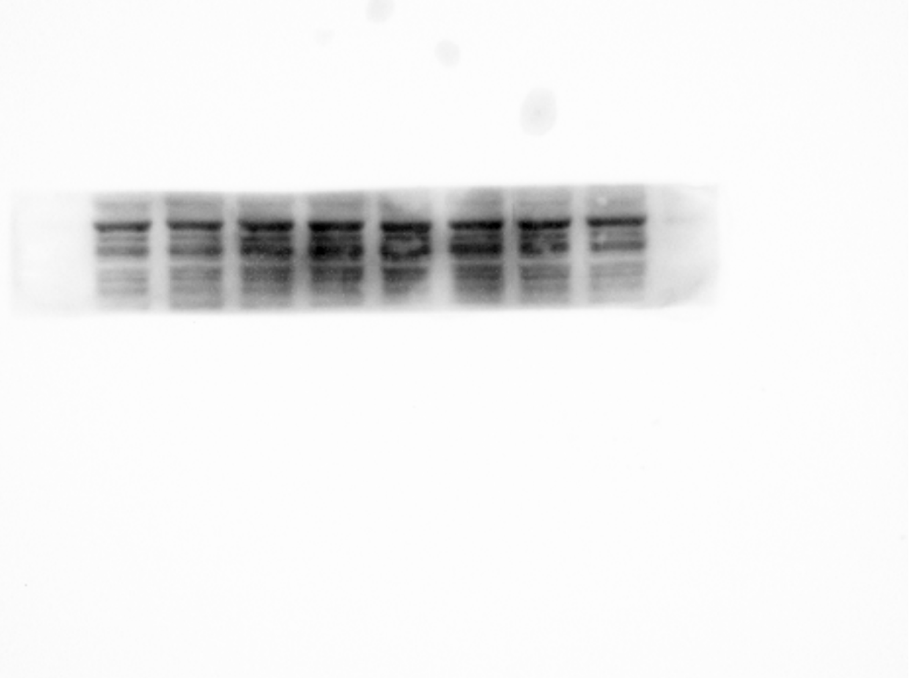


**CAA-CM**

**Control medium**
